# TNF Blockade Maintains an IL-10^+^ Phenotype in Human Effector CD4^+^ and CD8^+^ T Cells

**DOI:** 10.3389/fimmu.2017.00157

**Published:** 2017-02-15

**Authors:** Ceri A. Roberts, Lucy E. Durham, Veerle Fleskens, Hayley G. Evans, Leonie S. Taams

**Affiliations:** ^1^Division of Immunology, Infection and Inflammatory Disease (DIIID), Centre for Inflammation Biology and Cancer Immunology (CIBCI), King’s College London, London, UK

**Keywords:** tumor necrosis factor, anti-TNF, TNF inhibitors, adalimumab, interleukin-10, CD4^+^ T cell polarization, CD8^+^ T cell polarization, IL-10 regulation

## Abstract

CD4^+^ and CD8^+^ effector T cell subpopulations can display regulatory potential characterized by expression of the prototypically anti-inflammatory cytokine IL-10. However, the underlying cellular mechanisms that regulate expression of IL-10 in different T cell subpopulations are not yet fully elucidated. We recently showed that TNF inhibitors (TNFi) promote IL-10 expression in human CD4^+^ T cells, including IL-17^+^ CD4^+^ T cells. Here, we further characterized the regulation of IL-10 expression *via* blockade of TNF signaling or other cytokine/co-stimulatory pathways, in human T cell subpopulations. Addition of the TNFi drug adalimumab to anti-CD3-stimulated human CD4^+^ T cell/monocyte cocultures led to increased percentages of IL-10^+^ cells in pro-inflammatory IL-17^+^, IFNγ^+^, TNFα^+^, GM-CSF^+^, and IL-4^+^ CD4^+^ T cell subpopulations. Conversely, exogenous TNFα strongly decreased IL-10^+^ cell frequencies. TNF blockade also regulated IL-10 expression in CD4^+^ T cells upon antigenic stimulation. Using time course experiments in whole peripheral blood mononuclear cell (PBMC) cultures, we show that TNF blockade maintained, rather than increased, IL-10^+^ cell frequencies in both CD4^+^ and CD8^+^ T cells following *in vitro* stimulation in a dose- and time-dependent manner. Blockade of IL-17, IFNγ, IL-6R, or CD80/CD86-mediated co-stimulation did not significantly regulate IL-10 expression within CD4^+^ or CD8^+^ T cell subpopulations. We show that TNF blockade acts directly on effector CD4^+^ T cells, in the absence of monocytes or CD4^+^ CD25^high^CD127^low^ regulatory T cells and independently of IL-27, resulting in higher IL-10^+^ frequencies after 3 days in culture. IL-10/IL-10R blockade reduced the frequency of IL-10-expressing cells both in the presence and absence of TNF blockade. Addition of recombinant IL-10 alone was insufficient to drive an increase in IL-10^+^ CD4^+^ T cell frequencies in 3-day CD4^+^ T cell/monocyte cocultures, but resulted in increased IL-10 expression at later time points in whole PBMC cultures. Together, these data provide additional insights into the regulation of IL-10 expression in human T cells by TNF blockade. The maintenance of an IL-10^+^ phenotype across a broad range of effector T cell subsets may represent an underappreciated mechanism of action underlying this widely used therapeutic strategy.

## Introduction

The treatment of immune-mediated inflammatory diseases has improved considerably over the last 20 years with the advent of biological therapeutics. TNFα was the first cytokine to be fully validated as a therapeutic target in RA (1). TNFα inhibitors (TNFi) have revolutionized treatment of RA and have been used in over a million patients worldwide (2). Despite the good clinical response observed in many patients, TNFα blockade does not offer a curative treatment; approximately one-third of patients do not respond and loss of efficacy is frequently observed (3). Importantly, it is currently not possible to predict which patients will respond to TNFi therapy. In addition, in some inflammatory diseases such as Sjögren’s syndrome (4, 5) and multiple sclerosis (6), TNFi have not shown clinical efficacy. Furthermore, and paradoxically, some patients treated with TNFi develop *de novo* autoimmune diseases (7). These observations indicate that the underlying mechanisms relating to TNF blockade in humans are incompletely understood and require further exploration. The effects of TNFi are more wide-ranging than simply neutralizing the biological activity of soluble and membrane-bound TNFα (mTNFα). For example, by binding mTNFα, anti-TNF mAbs can mediate cell death by complement-dependent cytotoxicity and antibody-dependent cellular cytotoxicity (8–11). TNFα inhibitors have also been shown to affect downstream cytokine pathways (IL-1, IL-6, and IL-8) (2), modulate APC function (12), and promote regulatory T cell (Treg) expansion (13–15) although opposite findings regarding the latter have been reported (16–19).

Recent data from our laboratory demonstrated that TNF blockade promotes IL-10 expression in human CD4^+^ T cells (20). It was shown both cross-sectionally and longitudinally that inflammatory arthritis patients on TNFi therapy have an increased frequency of peripheral blood (PB) IL-10^+^ CD4^+^ T cells. These *in vivo* findings were reproduced *in vitro* by coculturing CD4^+^ T cells from healthy donors with autologous CD14^+^ monocytes and anti-CD3 mAb, in the presence of different TNFi drugs (adalimumab, infliximab, etanercept, or certolizumab) (20). Furthermore, we showed an increase in the percentage of IL-10 co-expressing IL-17^+^ CD4^+^ T cells, suggesting that otherwise pro-inflammatory cells displayed anti-inflammatory potential. Indeed, re-sorted TNFi-exposed IL-17^+^ CD4^+^ T cells secreted increased levels of IL-10, which was biologically active and could modulate markers of monocyte activation (20).

Although IL-17^+^ CD4^+^ T cells are recognized as an important cell population in inflammatory disease, other CD4^+^ T cell subsets also contribute to inflammation (21–24), as well as CD8^+^ T cells which can also be potent producers of pro-inflammatory cytokines (25–29). In this study, we therefore investigated *in vitro* whether TNF blockade regulates IL-10 expression in other pro-inflammatory cytokine-producing T cell subsets, whether blockade of other cytokines or T cell activation pathways also drives IL-10 expression, and how TNF blockade may manifest its IL-10-regulating effect on T cells.

## Materials and Methods

### Cell Isolation

Peripheral blood samples were obtained from healthy adult volunteers. Peripheral blood mononuclear cells (PBMCs) were isolated by density gradient centrifugation using Lymphoprep™ (Axis-Shield, Oslo, Norway). CD14^+^ monocytes and CD4^+^ T cells were isolated by magnetic-activated cell sorting (MACS) according to the manufacturer’s instructions (Miltenyi Biotec, Bergisch-Gladbach, Germany), and purity was confirmed by flow cytometry. Monocytes (average purity 98%) were isolated by positive selection using anti-CD14 microbeads. CD4^+^ T cells were isolated *via* negative depletion (average purity 95%), and in some experiments, CD45RO^+^ CD4^+^ T cells were subsequently enriched by positive selection using CD45RO microbeads (average purity 87%). In some experiments, CD4^+^ T cells were sorted to very high purity (> 99%) and part of the cells depleted of CD4^+^ CD25^high^CD127^low^ Tregs by FACS-sorting after labeling cells with CD4 PerCP Cy5.5 (SK3), CD25 PE (M-A251), CD127 Alexa Fluor 488 (A019D5) mAbs (all from BioLegend, Cambridge, UK). The study was approved by the Bromley Research Ethics Committee (06/Q0705/20), and written informed consent was obtained from all participants.

### Cell Culture

Cells were cultured at 37°C with 5% CO_2_ in culture medium [RPMI 1640 medium supplemented with 10% heat-inactivated fetal bovine serum (lot# 07F7435K, South American origin)] and 1% penicillin, streptomycin, and l-glutamine (all Life Technologies, Carlsbad, CA, USA). Freshly isolated bulk CD4^+^ or memory (CD45RO^+^)-enriched T cells (0.5 × 10^6^) and CD14^+^ monocytes (0.5 × 10^6^ unless otherwise indicated) were cocultured with 100 ng/ml anti-CD3 mAb (clone OKT3, Janssen-Cilag Ltd., High Wycombe, UK). Cocultures were incubated at 37°C with 5% CO_2_ for 3 days. In some experiments, MACS-isolated CD4^+^ T cells were cultured alone with anti-CD3/CD28 stimulation. Anti-CD3 (OKT3) was coated onto culture plates at 1.25 µg/ml in PBS for a minimum of 2 h at 37°C. Wells were then washed three times with PBS before adding CD4^+^ T cells in culture medium. Anti-CD28 (clone CD8.2; BD Biosciences) was added to the well at a final concentration of 1 µg/ml. In some experiments, whole PBMCs were cultured with 100 ng/ml anti-CD3 mAb for up to 5 days. Where indicated, the following recombinant cytokines, neutralizing antibodies, or other reagents (from R&D Systems unless otherwise indicated) were added at the start of the culture period: recombinant human (rh) TNFα (10 ng/ml; Biosource, Camarillo, CA, USA), rhIL-27 (10–100 ng/ml), rhIL-10 (10 ng/ml; rhIL-10 used to generate data in Figure S5 in Supplementary Material from PeproTech, Rocky Hill, NJ, USA), neutralizing/blocking antibodies to IFNγ (clone 25718, 10 µg/ml), IL-17 (clone 41809, 10 µg/ml), IL-1R1 (polyclonal, 2 µg/ml), IL-10 (clone 23738, 10 µg/ml), IL-10R (clone 8516, 10 µg/ml), and IL-27 (polyclonal, 5 µg/ml). The following isotype control antibodies were used at an assay-appropriate concentration: human IgG1 (Abcam, Cambridge, UK), mouse IgG1, mouse IgG2a, mouse IgG2b, goat IgG (all R&D Systems). Clinical grade biologics were purchased *via* Guy’s Hospital Pharmacy. Adalimumab (Abbott Laboratories, Chicago, IL, USA) was frozen in aliquots at −20°C and used at final concentration of 1 µg/ml unless otherwise indicated, tocilizumab (Roche, Welwyn Garden City, UK) was used at 50 µg/ml, and abatacept (Bristol-Myers Squibb, New York, NY, USA) was used at 5 µg/ml. Pediacel [Diphtheria, tetanus, pertussis (acellular, component), poliomyelitis (inactivated), and Haemophilus type b conjugate vaccine] and Revaxis [Diphtheria, tetanus and poliomyelitis (inactivated) vaccine] (gifts of James Reading, Tim Tree lab, King’s College London) were added at a 1:1,000 dilution.

### Flow Cytometry

To assess intracellular cytokine expression after cell culture, cells were restimulated for 3 h in the presence of phorbol 12-myristate 13-acetate (PMA; 50 ng/ml, Sigma-Aldrich), ionomycin (750 ng/ml, Sigma-Aldrich), and GolgiStop (according to the manufacturer’s instructions; BD Biosciences). In some experiments, cells were labeled with a fixable viability dye (eFluor506 or eFluor780, eBioscience, San Diego, CA, USA; LIVE/DEAD fixable dead cell stains, ThermoFisher Scientific) according to manufacturer’s instructions. Cells from cocultures were surface stained using anti-CD14 APC-Cy7 (HCD14; BioLegend) or anti-CD14 APC Vio770 (TUK4; Miltenyi Biotec) and in some experiments, anti-CD8 Pacific Blue (RPA-T8), before fixing in 2% paraformaldehyde (Merck & Co., Inc., Kenilworth, NJ, USA). Cells were washed and then permeabilized with 0.5% saponin (Sigma-Aldrich) and intracellularly stained with various combinations of the following fluorescently conjugated antibodies (all from BioLegend): anti-CD3 PE Cy7 (UCHT1), anti-CD4 PerCP Cy5.5 or Pacific Blue (both SK3), anti-IL-10 Alexa Fluor 488 (JES3-9D7), anti-IL-17 PE (BL168), anti-IFNγ PerCP Cy5.5, Pacific Blue or APC (all 4 S.B3), anti-TNFα APC (MAb11), GM-CSF APC (BVD2-21C11). Antibodies to detect CD4 and/or CD3 were added intracellularly, to allow for staining of these markers following our T cell culture conditions. Stained cells were acquired using a FACSCantoII or LSRFortessa (BD Biosciences); in most experiments, 100,000 T cell events were recorded. The gating strategies used to analyze intracellular cytokine expression in CD4^+^ T cells are described in Figure S1 in Supplementary Material. All flow cytometry data were analyzed using FlowJo software (version 7.6.1 or 10, Tree Star, Inc., Ashland, OR, USA).

### Enzyme-Linked Immunosorbent Assay (ELISA)

IL-10 was measured in cell culture supernatants using the IL-10 ELISA MAX kit (BioLegend), according to manufacturer’s instructions. IL-27 was measured in cell culture supernatants using the DuoSet Human IL-27 ELISA (eBioscience), according to manufacturer’s instructions. Microwell absorbance was read at 450 nm using a Wallac 1420 microplate reader (Perkin Elmer, Waltham, MA, USA). Concentrations of the analytes were determined based on the standard curve included on each plate.

### Statistical Analysis

Statistical testing was performed with GraphPad Prism 5.0 or 6.0 (GraphPad, San Diego, CA, USA). Data sets were tested for normality using the D’Agostino and Pearson omnibus normality test, followed by statistical significance testing using the appropriate tests as indicated in figure legends. Data sets with *n* values <8 were tested non-parametrically. *P* values < 0.05 were considered statistically significant.

## Results

### TNF Blockade Regulates IL-10 Expression in Human CD4^+^ and CD8^+^ T Cells

**Figure 1:**
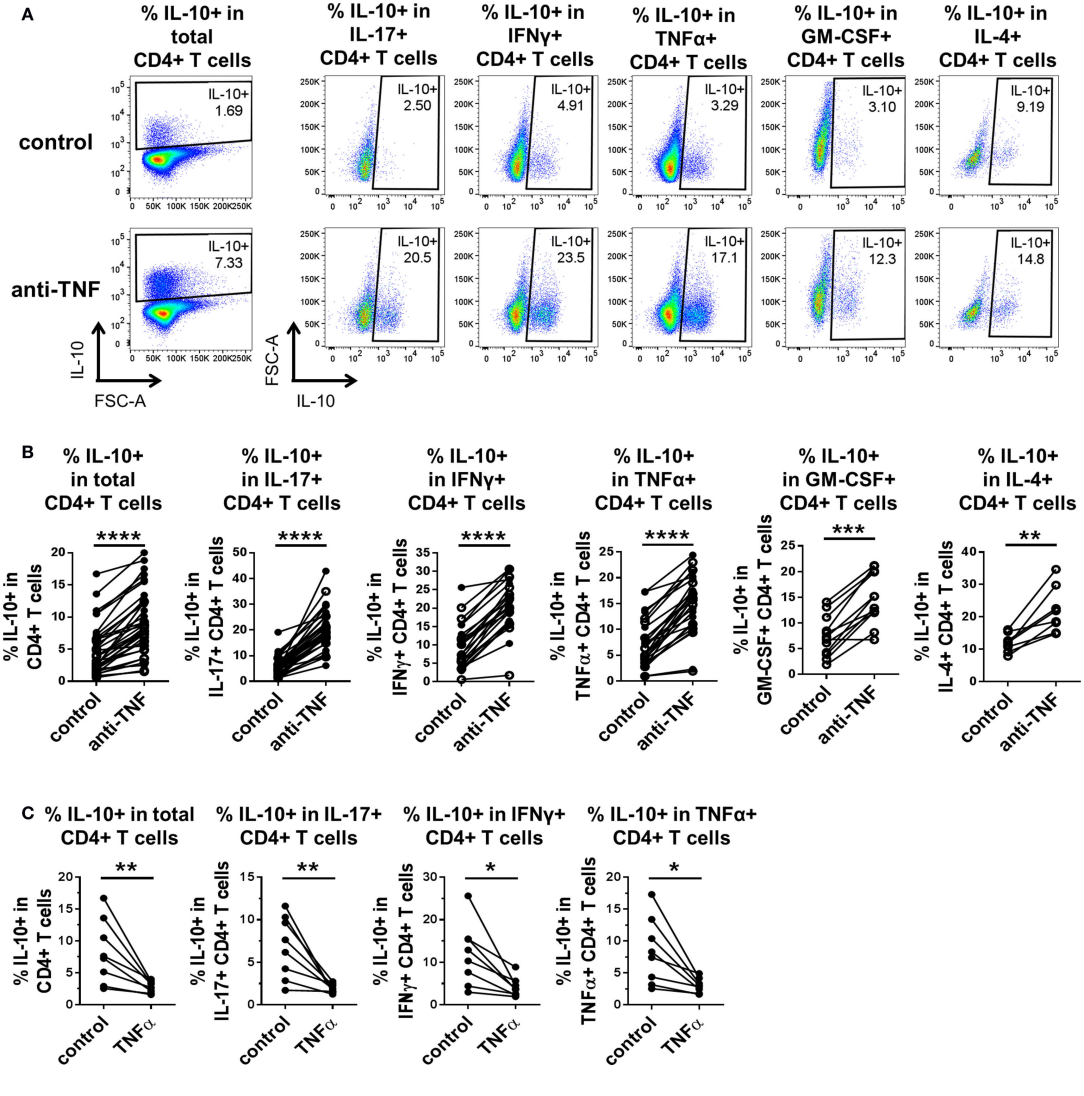
**TNF blockade promotes, while TNFα impairs IL-10 expression in pro-inflammatory CD4^+^ T cell subsets**. CD4^+^ T cells (open symbols) or CD4^+^ CD45RO^+^ T cells (filled symbols) were cocultured with autologous CD14^+^ monocytes and anti-CD3 mAb (100 ng/ml) in the absence or presence of the anti-TNF mAb adalimumab (1 µg/ml) **(A,B)** or rhTNFα (10 ng/ml) **(C)**. After 3 days, cells were restimulated and assessed for intracellular cytokine expression as described in the methods. CD4^+^ T cells were gated as described in Figure S1 in Supplementary Material. **(A,B)** Representative dot plots **(A)** and cumulative data **(B)** showing the percentages of IL-10^+^ cells within total CD4^+^ T cells (*n* = 35), or within IL-17^+^ CD4^+^ (*n* = 35), IFNγ^+^ CD4^+^ (*n* = 24), TNFα^+^ CD4^+^ (*n* = 27), GM-CSF^+^ CD4^+^ (*n* = 12), or IL-4^+^ CD4^+^ (*n* = 8) cells after culture in the absence or presence of anti-TNF. Data were analyzed using Wilcoxon matched-pairs signed rank test. **(C)** Cumulative data showing the percentages of IL-10^+^ cells within total CD4^+^ CD45RO^+^ T cells or within IL-17^+^ (*n* = 8), IFNγ^+^ (*n* = 8), or TNFα^+^ (*n* = 8) CD4^+^ CD45RO^+^ T cells after culture in the absence or presence of rhTNFα. Data were analyzed using paired *t* test. Each connecting line represents an individual donor (*p < 0.05, **p < 0.01, ***p < 0.001, ****p < 0.0001).

To investigate whether TNF blockade regulates IL-10 expression in different pro-inflammatory cytokine-producing CD4^+^ T cells, we isolated CD4^+^ T cells from PB of healthy donors and cocultured the cells with CD14^+^ monocytes and anti-CD3 mAb (100 ng/ml) in the absence or presence of the anti-TNF mAb adalimumab (1 µg/ml), as previously described (20). After 3 days, cells were restimulated with PMA and ionomycin in the presence of GolgiStop for 3 h and stained for cytokine expression (gating strategy shown in Figure S1 in Supplementary Material). In agreement with our previous data (20), TNF blockade led to a significant increase in IL-10^+^ cells within total CD4^+^ T cells as well as in the IL-17^+^ CD4^+^ T cell subset. In addition, we found a strong increase in IL-10-expressing cells within IFNγ^+^, TNFα^+^, GM-CSF^+^, and IL-4^+^ CD4^+^ T cell populations (Figures 1A,B). TNF blockade did not alter the frequencies of IFNγ^+^, TNFα^+^, or GM-CSF^+^ CD4^+^ T cell populations but did induce a modest increase in IL-17^+^ CD4^+^ T cells, as previously shown (20), and in most donors in IL-4^+^ frequencies, although this did not reach statistical significance (Figures S2A, B in Supplementary Material). Addition of an isotype control human IgG1 mAb did not promote IL-10 expression in CD4^+^ T cells (Figure S3 in Supplementary Material).

Contrary to the effects of TNF blockade, addition of rhTNFα to cocultures of CD4^+^ CD45RO^+^ T cells, monocytes and anti-CD3 mAb led to a striking decrease in the percentage of IL-10^+^ cells within total CD4^+^ T cells and within IL-17^+^, IFNγ^+^, and TNFα^+^ CD4^+^ T cell subsets (GM-CSF^+^ and IL-4^+^ CD4^+^ T cells were not tested) (Figure 1C). TNFα addition did not significantly alter the frequencies of IL-17^+^, IFNγ^+^, or TNFα^+^ CD4^+^ T cell subsets (Figure S2C in Supplementary Material).

**Figure 2:**
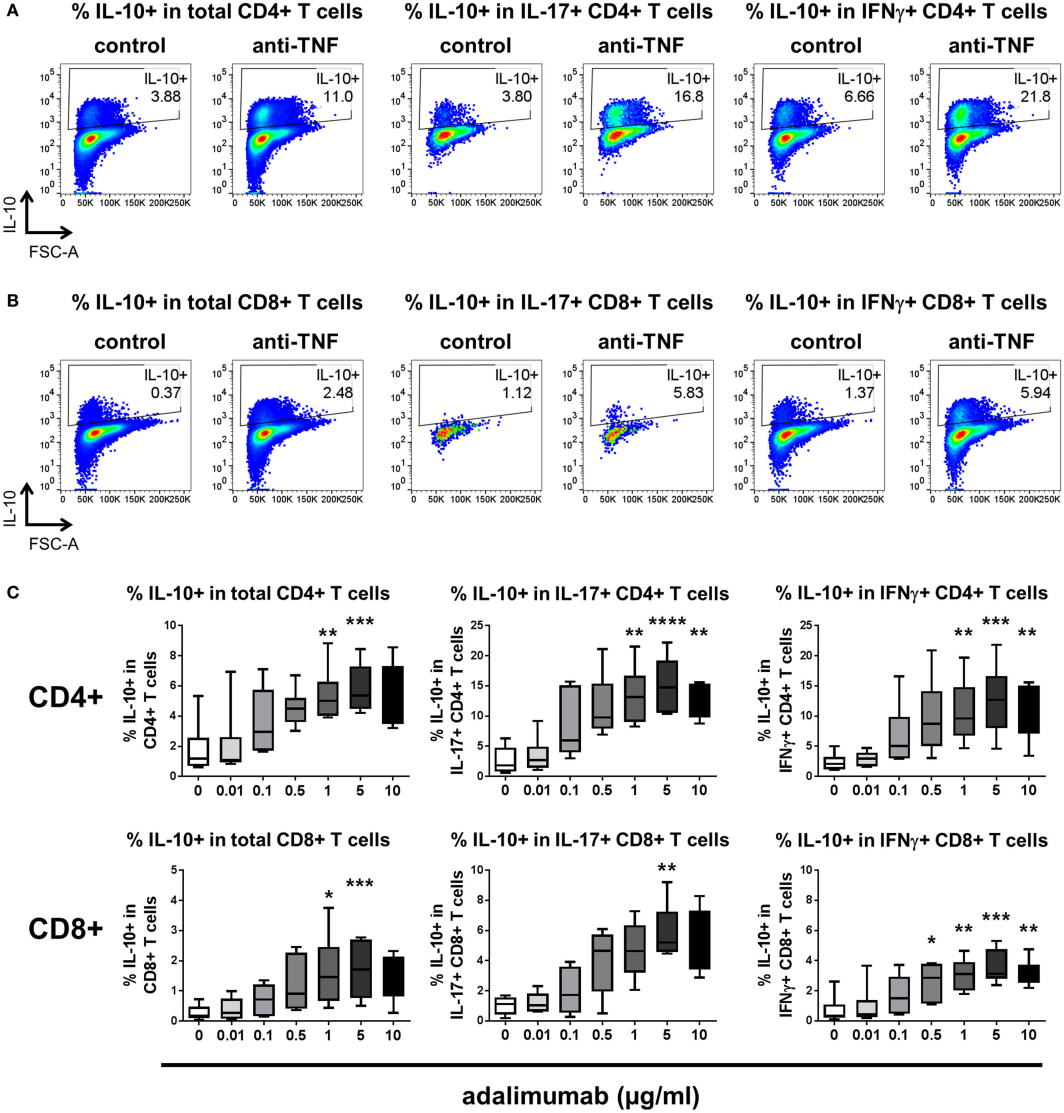
**TNF blockade regulates IL-10 expression in CD4^+^ and CD8^+^ T cells in a dose-dependent manner**. **(A,B)** Freshly isolated peripheral blood mononuclear cells were cultured with anti-CD3 mAb (100 ng/ml) in the absence (control) or presence (anti-TNF) of adalimumab (1 µg/ml). After 3 days, cells were restimulated and assessed for intracellular cytokine expression as described in the methods. Cells were gated on live single CD3^+^ cells and IL-10 expression was assessed within CD4^+^ **(A)** or CD8^+^ **(B)** T cell subsets. Representative dot plots show the percentages of IL-10^+^ cells within total CD4^+^ or CD8^+^ T cells, or within IL-17^+^ or IFNγ^+^ subsets after culture in the absence or presence of anti-TNF. **(C)** Cells were cultured as described above in the presence of increasing doses of adalimumab (0–10 μg/ml). Box-whisker plots represent data from *n* = 6 individual donors; whiskers show minimum to maximum values. Data were analyzed by Friedman test with comparison to control by Dunn’s multiple comparisons test (*p < 0.05, **p < 0.01, ***p < 0.001, ****p < 0.0001).

Since pro-inflammatory cytokine-expressing CD8^+^ T cells also contribute to immune-mediated inflammatory diseases (27–29), we investigated whether TNF blockade can regulate IL-10 expression in CD8^+^ T cells. We adapted the culture system to stimulate whole PBMC with anti-CD3 mAb (100 ng/ml) in the absence or presence of a dose range of adalimumab (0.01–10 µg/ml). After 3 days, the cultures were restimulated with PMA/ionomycin in the presence of Golgistop and cytokine expression was assessed within either the CD4^+^ or the CD8^+^ T cell populations. Significant increases in the percentages of IL-10^+^ cells within both CD4^+^ and CD8^+^ T cell populations were observed, including in the IL-17^+^ and IFNγ^+^ subpopulations (Figure 2). The regulation of IL-10 expression by TNF blockade was dose responsive in both CD4^+^ and CD8^+^ T cell subsets, with significantly increased IL-10^+^ frequencies following culture with either 1 or 5 µg/ml adalimumab (Figure 2C). These doses reflect the clinically effective trough levels for adalimumab in human serum following treatment (30, 31). In agreement with data from the CD4^+^ T cell/monocyte cocultures, addition of rhTNFα (100 ng/ml) to anti-CD3-stimulated PBMC led to a reduction in IL-10^+^ cell frequencies, in both CD4^+^ and CD8^+^ T cells (*n* = 3, data not shown).

Since these *in vitro* studies of T cell responses to TNF blockade all relied on monoclonal anti-CD3 stimulation, we tested whether TNF blockade could still elicit increased IL-10^+^ T cell responses in the context of a more physiological antigenic stimulation. Pediacel is a pentavalent vaccine consisting of purified diphtheria toxoid, purified tetanus toxoid, acellular pertussis vaccine, inactivated poliovirus, and *Haemophilus influenzae* type b polysaccharide. Revaxis is a booster vaccine containing purified diphtheria and tetanus toxoids and inactivated poliovirus. Each vaccine was added to CD4^+^ T cell/monocyte cocultures (1:1,000 dilution) in the presence or absence of TNF blockade. After 6 days, antigen-stimulated CD4^+^ T cells cultured in the presence of adalimumab demonstrated elevated IL-10^+^ frequencies as compared to cells that were not exposed to TNF blockade (Figure S4 in Supplementary Material).

Together these data demonstrate that *in vitro* TNF blockade provided at a physiological concentration and in a physiological setup can promote IL-10 expression in CD4^+^ and CD8^+^ T cells, including in subsets expressing pro-inflammatory cytokines.

### TNF Blockade Maintains IL-10 Expression in CD4^+^ and CD8^+^ T Cells

**Figure 3:**
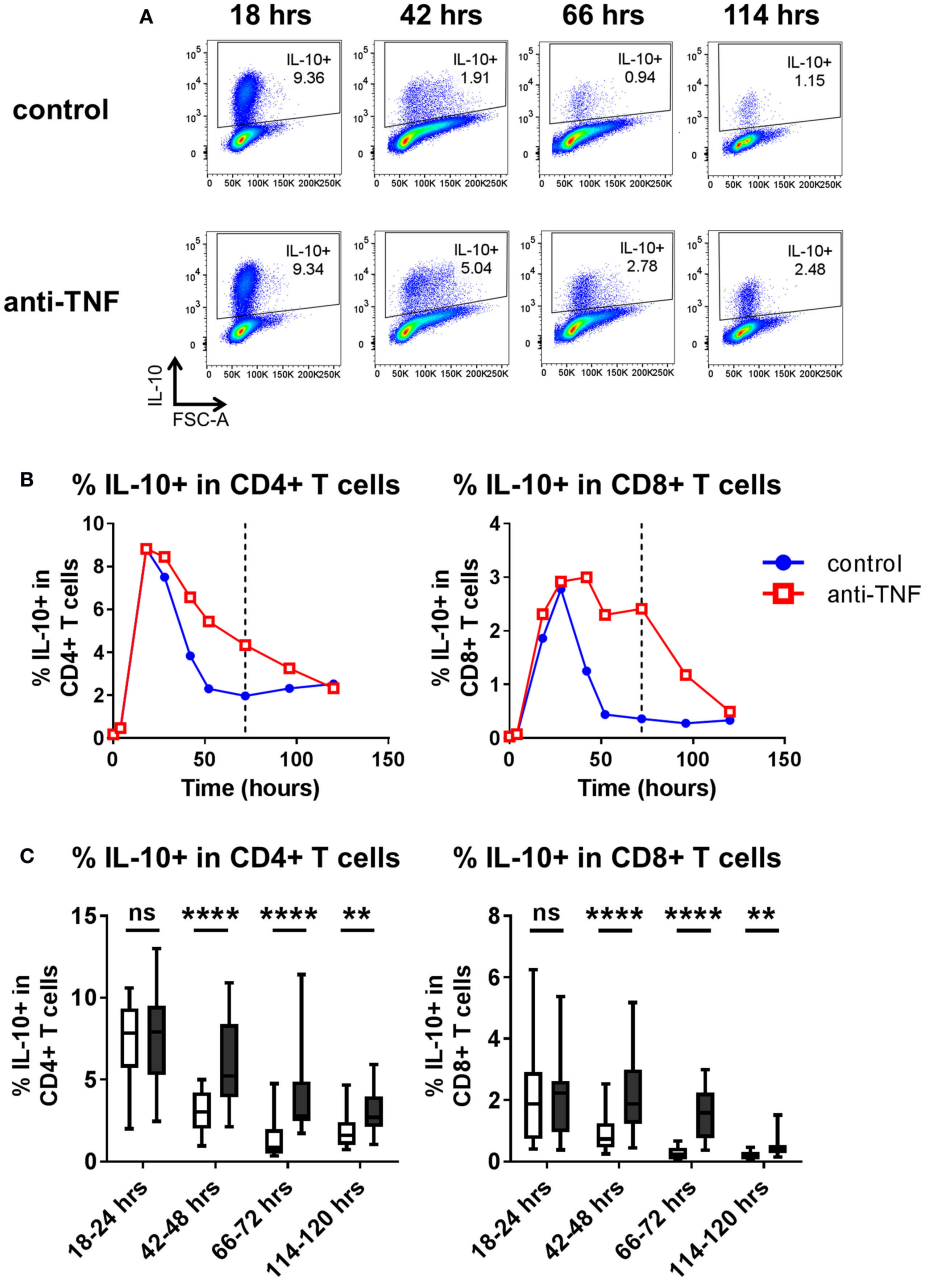
**Kinetics of IL-10 regulation by TNF blockade**. Peripheral blood mononuclear cells were cultured in the presence of anti-CD3 mAb (100 ng/ml), with or without anti-TNF (adalimumab, 1 μg/ml). At various time points (0–120 h), cells were restimulated and assessed for intracellular cytokine expression as described in the methods. **(A)** Representative dot plots show frequencies of IL-10^+^ cells within the total CD4^+^ T cell population 18, 42, 66, and 114 h after culture with or without anti-TNF. **(B)** Representative time course data demonstrating IL-10^+^ frequencies within CD4^+^ and CD8^+^ T cells in the presence (red lines) or absence (blue lines) of anti-TNF. **(C)** Box-whisker plots represent data from *n* = 15 individual donors showing the effect of TNF blockade on IL-10^+^ frequencies at 18–24 h, 42–48 h, and 66–72 h and *n* = 10 individual donors at 114–20 h; whiskers show minimum to maximum values. Each time point was analyzed by Wilcoxon matched-pairs signed rank test. ns *p* > 0.05, **p < 0.01, ****p < 0.0001.

Thus far, assessment of cytokine expression following *in vitro* TNF blockade was carried out after 3 days of culture. Time course experiments were performed to assess the kinetics of IL-10 expression in CD4^+^ and CD8^+^ T cells. PBMC were cultured with anti-CD3 mAb in the absence or presence of adalimumab, and the frequencies of IL-10 expressing cells were analyzed 4–120 h after stimulation. The data show that early after TCR stimulation (18–24 h) the frequencies of IL-10 expressing cells within CD4^+^ and CD8^+^ T cells increased rapidly irrespective of the presence of TNF blockade. However, at later time points (from 42 to 120 h), higher frequencies of IL-10^+^ cells were sustained within CD4^+^ and CD8^+^ T cells in the presence of TNF blockade (Figure 3). These experiments suggest that TNF blockade maintains, rather than directly induces, an IL-10^+^ phenotype in CD4^+^ and CD8^+^ T cells following TCR stimulation.

### Blockade of TNFα, But Not IL-17, IFNγ, IL-6R, or CD80/CD86-Mediated Co-Stimulation, Regulates IL-10 in Human CD4^+^ T Cells

**Figure 4:**
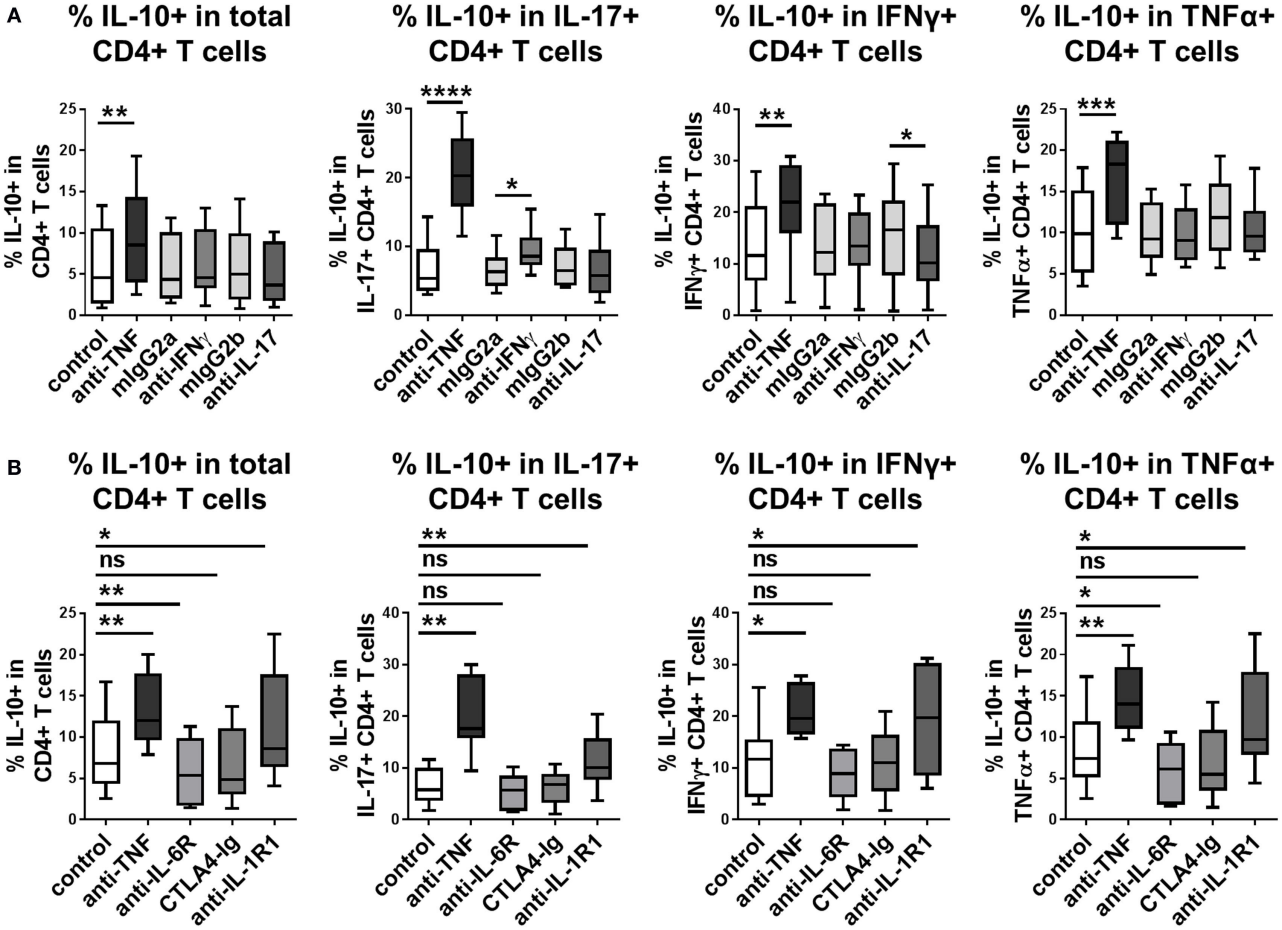
**Blockade of TNFα signaling, but not IL-17, IFNγ, IL-6R, or CD80/CD86-mediated co-stimulation, regulates IL-10 in human CD4^+^ T cells**. **(A)** CD4^+^ T cells (*n* = 6) or CD45RO^+^ enriched CD4^+^ T cells (*n* = 2) were cocultured 1:1 with autologous monocytes in the presence of anti-CD3 (100 ng/ml), without (control) or with anti-TNF mAb (adalimumab, 1 μg/ml) or neutralizing antibodies to IFNγ or IL-17 (10 μg/ml) or the appropriate isotype control antibodies (mIgG2a and mIgG2b, respectively). **(B)** CD45RO^+^ enriched CD4^+^ T cells (*n* = 9) were cocultured 1:1 with autologous monocytes in the presence of anti-CD3 (100 ng/ml), without (control) or with anti-TNF mAb (adalimumab, 1 μg/ml), tocilizumab (50 µg/ml), abatacept (5 µg/ml) or anti-IL-1R1-blocking Ab (2 μg/ml). **(A,B)** After 3 days, cells were restimulated and assessed for intracellular cytokine expression as described in the methods. Data show frequencies of IL-10^+^ cells within the total CD4^+^ T cell population or within IL-17^+^, IFNγ^+^, or TNFα^+^ CD4^+^ T cell subpopulations. Data were analyzed by Wilcoxon matched-pairs signed rank test with comparison to the appropriate control condition (ns *p* > 0.05, *p < 0.05, **p < 0.01, ***p < 0.001, ****p < 0.0001).

Our previous work demonstrated that in addition to adalimumab, other TNFα inhibitors (etanercept, infliximab, or certolizumab) as well as TNFR1/2 blocking mAbs were capable of increasing frequencies of IL-10-expressing IL-17^+^ CD4^+^ T cells (20). We investigated whether blockade of additional pro-inflammatory pathways could promote IL-10 expression in CD4^+^ T cells. Blockade of IL-17A did not enhance the frequencies of IL-10^+^ cells in any of the CD4^+^ T cell populations tested (Figure 4A). Blockade of IFNγ did not affect the percentage of IL-10^+^ cells within total CD4^+^ T cells, or within IFNγ or TNFα^+^ subpopulations, but led to modestly increased frequencies of IL-10^+^ expressing cells within the IL-17^+^ population, although this effect was much weaker than the effect of TNF blockade in parallel cultures (Figure 4A). Addition of tocilizumab (IL-6R blockade) or abatacept (CTLA4-Ig, which blocks CD80/CD86-mediated co-stimulation), both of which are biologic drugs routinely used in the clinic to treat rheumatoid arthritis, did not increase IL-10^+^ frequencies in CD4^+^ T cells, IL-17^+^, IFNγ^+^, or TNFα^+^ CD4^+^ T cell subpopulations (Figure 4B). To determine whether blockade of these pathways might regulate IL-10 expression with different kinetics to TNF blockade, IL-10^+^ frequencies were analyzed within both CD4^+^ and CD8^+^ T cells at different time points in anti-CD3-stimulated PBMC cultures exposed to these antibodies or drugs. IL-10^+^ CD4^+^ and IL-10^+^ CD8^+^ T cell frequencies were not regulated at any time point by blockade of IL-17, IFNγ, IL-6R, or CD80/CD86-mediated co-stimulation (Figure S5 in Supplementary Material). Blockade of IL-1R1 in CD4^+^ T cell/monocyte cocultures resulted in a significantly increased proportion of IL-10^+^ cells within total CD4^+^ T cells and within IL-17^+^, IFNγ^+^, or TNFα^+^ subpopulations (Figure 4B). However, this effect was not replicated in either CD4^+^ or CD8^+^ T cells in whole PBMC cultures (Figure S5 in Supplementary Material), indicating that the capacity of IL-1 blockade to regulate IL-10 expression may be dependent on the *in vitro* culture conditions. Together these data indicate that IL-10 expression in CD4^+^ T cells and CD8^+^ T cells can be regulated by blocking TNFα signaling, but not by blocking IFNγ, IL-17, IL-6R, or CD80/CD86-mediated co-stimulation, at least *in vitro*.

### TNFα Blockade Directly Regulates IL-10 Expression in Effector CD4^+^ T Cells in the Absence of Monocytes

**Figure 5:**
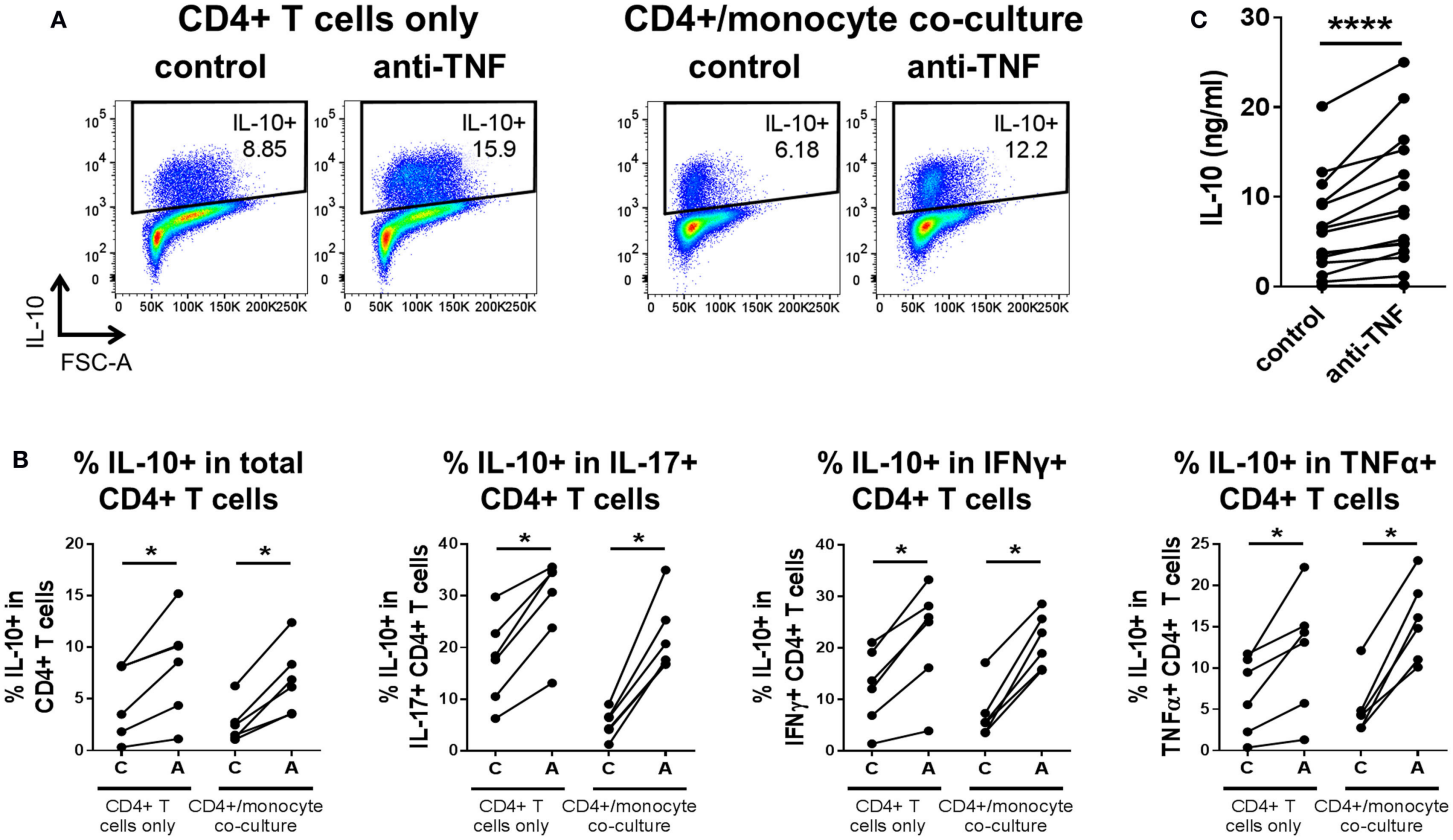
**TNF blockade directly regulates IL-10 expression in CD4^+^ T cells in the absence of monocytes**. CD4^+^ T cells were stimulated with immobilized anti-CD3 (1.25 μg/ml) and soluble CD28 (1 μg/ml), indicated as “CD4^+^ T cells only,” or were cocultured 1:1 with autologous monocytes and stimulated with soluble anti-CD3 (100 ng/ml), indicated as “CD4^+^/monocyte coculture.” These cultures were carried out in the absence [(C), control] or presence [(A), adalimumab] of anti-TNF mAb (1 μg/ml). **(A,B)** After 3 days, cells were restimulated and assessed for intracellular cytokine expression as described in the methods. **(A)** Representative dot plots show frequencies of IL-10^+^ cells within the total CD4^+^ T cell population in CD4^+^ T cells only or CD4^+^/monocyte coculture, with or without anti-TNF. **(B)** Cumulative data (*n* = 6) showing frequencies of IL-10^+^ cells within the total CD4^+^ T cell population or within IL-17^+^, IFNγ^+^ or TNFα^+^ CD4^+^ T cell subpopulations. **(C)** After 3 days in culture but prior to re-stimulation, supernatants were harvested from CD4^+^ T cell only cultures (*n* = 15) and analyzed by enzyme-linked immunosorbent assay for IL-10 secretion. Data were analyzed by Wilcoxon matched-pairs signed rank test (*p < 0.05, ****p < 0.0001).

Since CD14^+^ monocytes are major producers of TNFα, we explored whether the presence of monocytes was required for the effects of TNF blockade on regulating IL-10. CD4^+^ T cells were sorted to a very high purity (>99%) and stimulated with plate-bound anti-CD3 and soluble anti-CD28 mAb in the absence of monocytes or placed in coculture with monocytes and anti-CD3 mAb, with or without addition of anti-TNF mAb. In the CD4^+^ T cell only cultures, TNF blockade still brought about increased IL-10^+^ cell frequencies within the total CD4^+^ population and also within IL-17^+^, IFNγ^+^, or TNFα^+^ subpopulations, similar to the increased IL-10^+^ frequencies observed in CD4 T cell/monocyte cocultures (Figures 5A,B). Analysis of supernatants from anti-CD3/CD28-stimulated CD4^+^ T cells by ELISA confirmed that levels of secreted IL-10 were increased during culture in the presence of TNF blockade (Figure 5C).

**Figure 6:**
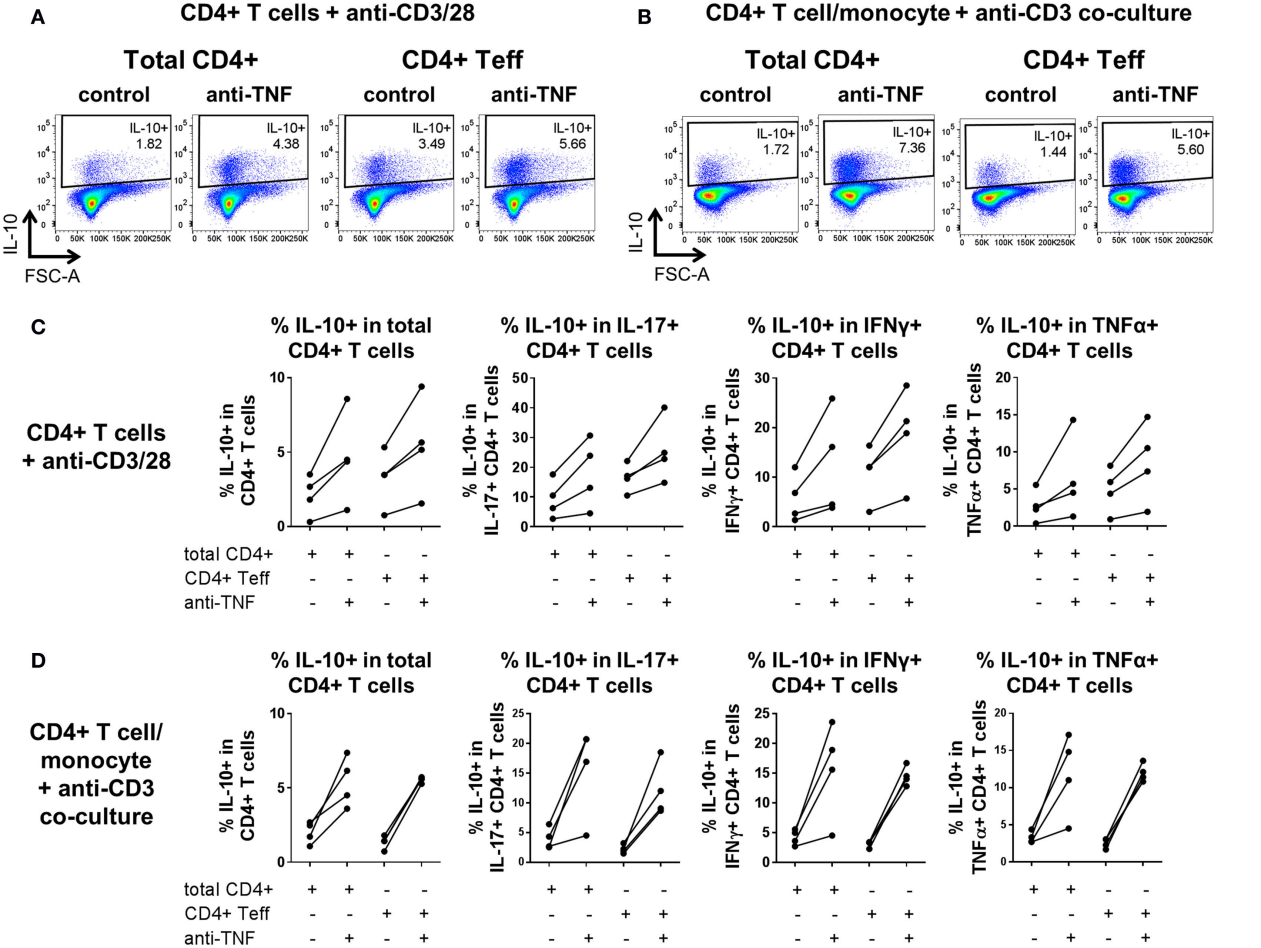
**TNF blockade increases IL-10^+^ cell frequencies in CD4^+^ effector T cells depleted of regulatory T cells (Tregs)**. MACS-isolated CD4^+^ T cells from four donors were FACS-sorted to high purity (>99%) to obtain total CD4^+^ cells (Total) or CD4^+^ effector T cells depleted of CD25^high^ CD17^low^ Tregs (Teff). Total/Teff CD4^+^ T cells were either stimulated with immobilized anti-CD3 (1.25 μg/ml) and soluble CD28 (1 μg/ml) or cocultured 1:1 with autologous monocytes and stimulated with soluble anti-CD3 (100 ng/ml), in the absence (control) or presence (anti-TNF) of adalimumab (1 μg/ml). After 3 days, cells were restimulated and assessed for intracellular cytokine expression as described in the methods. **(A,B)** Representative dot plots show frequencies of IL-10^+^ cells within the CD4^+^ T cell population in **(A)** anti-CD3/28-stimulated total/effector CD4^+^ T cells or **(B)** total/effector CD4^+^ T cell/monocyte cocultures, both with or without anti-TNF. **(C,D)** Cumulative data (*n* = 4) showing frequencies of IL-10^+^ cells within the CD4^+^ T cell population or within IL-17^+^, IFNγ^+^, or TNFα^+^ CD4^+^ T cell subpopulations, in either **(C)** anti-CD3/28-stimulated total/effector CD4^+^ T cells or **(D)** total/effector CD4^+^ T cell/monocyte cocultures, both with or without anti-TNF.

One potential interpretation of the observed increased frequencies of IL-10^+^ cells following TNF blockade could be that there is an expansion of a pre-existing population of Tregs. Our previous work indicated that in three donors, MACS-depletion of CD4^+^ CD25^+^ cells did not impair the anti-TNF-mediated increase in IL-10^+^ frequencies within IL-17^+^ CD4^+^ T cells and that no increase in FOXP3^+^ cell frequencies was apparent upon TNF blockade (20). To investigate this further, MACS-isolated CD4^+^ T cells were FACS sorted to very high purity (>99%) to yield either total CD4^+^ T cells or effector CD4^+^ CD25-CD127^+^ T cells (Teff; depleted of CD25^high^ CD127^low^ Treg). These cells were then cultured with anti-CD3/CD28 mAbs in the absence of monocytes, or placed in coculture with monocytes and anti-CD3 mAb, in the absence or presence of adalimumab. Our data show that TNF blockade still resulted in increased frequencies of IL-10-expressing cells in effector T cells in the absence of CD25^high^ CD127^low^ Tregs (Figure 6). Together these data indicate that TNF blockade regulates IL-10 expression directly in CD4^+^ effector T cells and does not require the presence of CD14^+^ monocytes or CD4^+^ CD25^high^CD127^low^ Tregs.

In support of our findings that TNF blockade can exert its effects on CD4^+^ T cells in the absence of monocytes, we did not find evidence for a role of the monocyte-derived anti-inflammatory mediator IL-27 (32) in mediating IL-10 expression in the context of TNF blockade. This was demonstrated by the findings that TNF blockade did not induce IL-27 secretion, rhIL-27 enhanced IL-10 secretion but did not result in increased percentages of IL-10^+^ CD4^+^ T cells, and IL-27 blockade did not impair the increased IL-10^+^ CD4^+^ T cell frequencies brought about by TNF blockade (Figure S6 in Supplementary Material).

### IL-10 Partly Contributes to TNF Blockade-Mediated IL-10 Regulation

**Figure 7:**
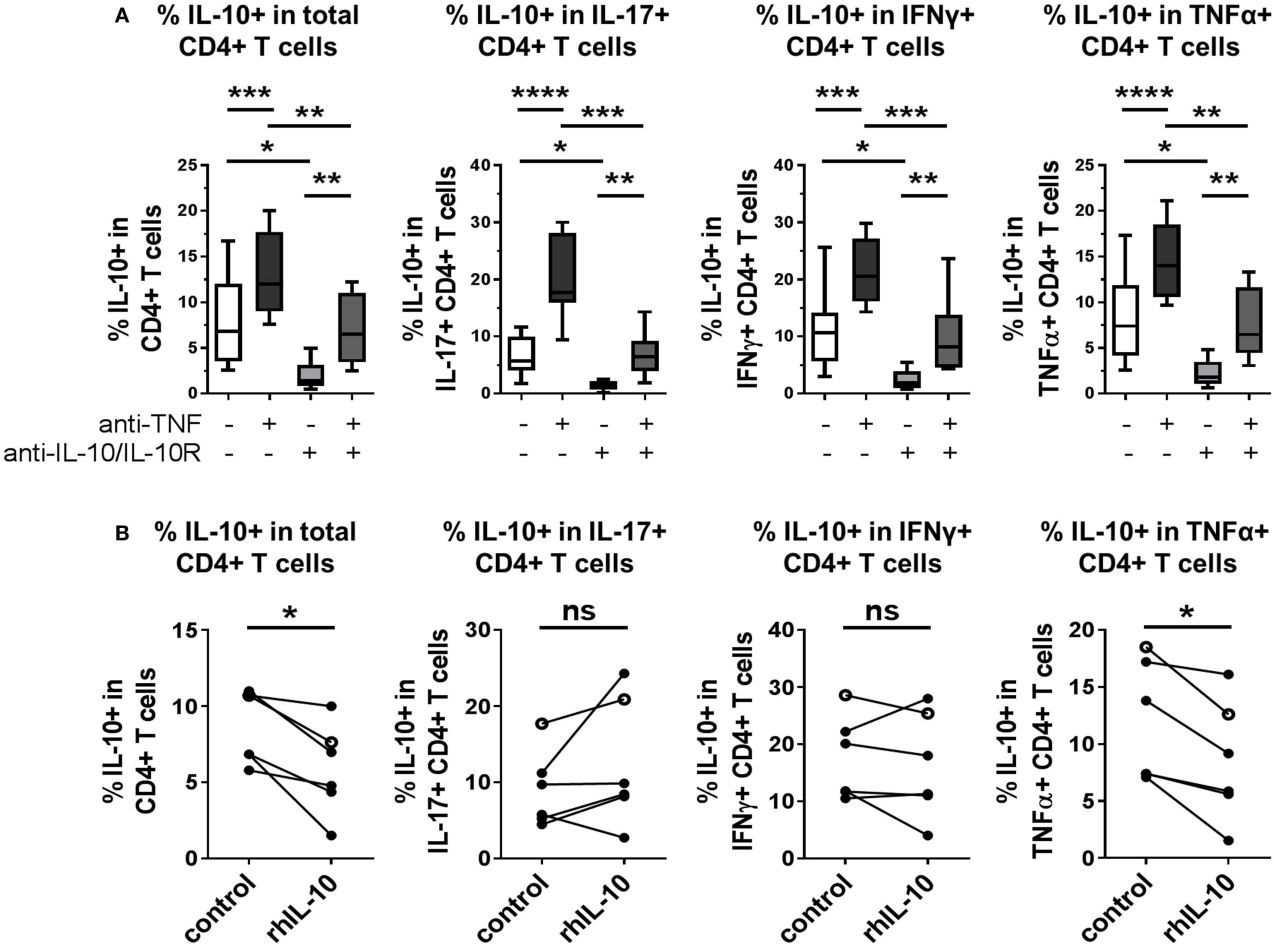
**IL-10 expression in CD4^+^ T cells is inhibited by IL-10 blockade, in both the absence and presence of TNF blockade**. **(A)** CD45RO^+^ enriched CD4^+^ T cells were cocultured 1:1 with autologous monocytes in the presence of anti-CD3 (OKT3,100 ng/ml), with or without anti-TNF mAb (adalimumab, 1 μg/ml), with or without neutralizing antibodies to IL-10 and IL-10R (both 10 μg/ml). After 3 days, cells were restimulated and assessed for intracellular cytokine expression as described in the methods. Box-whisker plots show frequency of IL-10^+^ cells within the total CD4^+^ T cell population or within IL-17^+^, IFNγ^+^ or TNFα^+^ subpopulations; whiskers show minimum to maximum values. Data were analyzed by repeated measures ANOVA, with comparison between selected conditions by Sidak’s multiple comparison test (*n* = 9). **(B)** CD45RO^+^ enriched (filled symbols, *n* = 5) or bulk (open symbols, *n* = 1) CD4^+^ T cells were cocultured 1:1 with autologous monocytes and stimulated with anti-CD3 mAb (100 ng/ml), with or without recombinant human IL-10 (rhIL-10; 10 ng/ml). After 3 days, cells were restimulated and assessed for intracellular cytokine expression as described in the methods. Data show frequencies of IL-10^+^ cells within the total CD4^+^ T cell population or within IL-17^+^, IFNγ^+^, or TNFα^+^ subpopulations and were assessed by Wilcoxon matched-pairs signed rank test (ns *p* > 0.05, *p < 0.05, **p < 0.01, ***p < 0.001, ****p < 0.0001).

Finally, we explored whether modulation of IL-10 expression in CD4^+^ T cells by TNF blockade was dependent on IL-10 signaling itself. Neutralizing antibodies against IL-10 and IL-10R were added to CD4^+^ T cell/monocyte cocultures stimulated with anti-CD3 mAb, in the presence or absence of TNF blockade, and intracellular cytokine expression was assessed after 3 days. Blockade of IL-10 signaling in the absence of adalimumab led to significantly reduced IL-10^+^ frequencies within total CD4^+^ T cells and within IL-17^+^, IFNγ, or TNFα^+^ subpopulations (Figure 7A). When adalimumab and IL-10/IL-10R blocking mAbs were added in combination to the cocultures, a decrease in IL-10^+^ CD4^+^ T cell frequencies was observed as compared to the condition treated with adalimumab alone; however, an increase was still observed as compared to IL-10/IL-10R blockade alone. These data suggest that pathways additional to IL-10 signaling could play a role in the regulation of IL-10 expression by TNF blockade. Indeed, when we tested whether addition of exogenous IL-10 (10 ng/ml) could drive IL-10 expression in CD4^+^ T cell/monocyte cocultures, we found that this by itself was not sufficient to increase IL-10^+^ cell frequencies in total CD4^+^ T cells, or within IL-17^+^, IFNγ^+^, or TNFα^+^ subpopulations. Addition of rhIL-10 even decreased the percentage of IL-10^+^ cells in total CD4^+^ T cells and TNFα^+^ CD4^+^ T cells (Figure 7B). The biological activity of the recombinant IL-10 was validated by culturing freshly isolated monocytes with 0, 10, or 100 ng/ml rhIL-10 for 20 h and confirming that rhIL-10 addition resulted in increased CD14 and CD163 and reduced HLA-DR expression by flow cytometry, in accordance with previous studies (33, 34) (*n* = 2, data not shown). We next investigated the regulation of IL-10 in response to rhIL-10 in both CD4^+^ and CD8^+^ T cells using whole PBMC cultures assessed for IL-10 expression over a 5-day time course (Figure S5 in Supplementary Material). We found that at earlier time points (18–24 h), rhIL-10 addition reduced IL-10^+^ CD4^+^ T cell frequencies (*n* = 6, *p* = 0.03, Wilcoxon matched-pairs signed rank test), while at later time points (66–72 and 114–120 h), rhIL-10 slightly increased IL-10^+^ CD4^+^ T cell frequencies (*n* = 6, *p* = 0.03, Wilcoxon matched-pairs signed rank test). IL-10^+^ CD8^+^ T cell frequencies remained very low throughout, regardless of rhIL-10 addition. Together, these data indicate that IL-10 signaling contributes in part to TNF blockade-mediated regulation of IL-10 expression in CD4^+^ T cells, but that other factors also play a role. The effects of exogenous IL-10 on regulating IL-10 expression in CD4^+^ T cells appear to be dependent on both time and *in vitro* culture conditions.

## Discussion

Here, we show that T cell stimulation in the presence of TNF blockade maintains the proportion of cells expressing the anti-inflammatory cytokine IL-10. This phenomenon is observed in total CD4^+^ and CD8^+^ T cells as well as within a variety of pro-inflammatory cytokine-expressing (IL-17^+^, IFNγ^+^, TNFα^+^, GM-CSF^+^ or IL-4^+^) subpopulations. TNF blockade regulates IL-10 expression whether CD4^+^ T cells are stimulated in the presence or absence of monocytes, and when an antigenic stimulus is used in place of monoclonal anti-CD3 stimulation. We found that blockade of IL-17, IFNγ, IL-6R, or CD80/CD86-mediated co-stimulation did not regulate IL-10 expression in CD4^+^ or CD8^+^ T cell subpopulations. Blocking antibodies to IL-1R1 resulted in increased IL-10^+^ CD4^+^ T cell frequencies when added to CD4^+^ T cell/monocyte cocultures, but this effect was not replicated in whole PBMC cultures. One explanation could be that additional cell types are present in PBMC cultures that can produce or respond to IL-1. Neither CD4^+^ CD25^high^CD127^low^ Tregs nor IL-27 was required for TNF blockade to exert its IL-10-promoting effects.

Our data confirm and extend our previous work showing that TNF blockade promotes IL-10 expression in CD4^+^ T cells (20) and support other findings in the literature. A small study of RA patients found that frequencies of IL-10^+^ PBMC were increased following treatment with infliximab (14). However, in the three patients studied, it was not confirmed which cell subset(s) expressed IL-10. Ebert later showed that supernatants from cultures of either monocytes (adherent PBMC) or T cells (non-adherent, CD14^−^ CD20^−^ HLA-DR^−^ PBMC), contained increased IL-10 following exposure to infliximab *in vitro* (35). Antiga et al. demonstrated increased percentages of IL-10^+^ CD4^+^ T cells in T cell blasts isolated from skin lesions of psoriasis patients, following treatment with etanercept (36), and PB IL-10^+^ CD4^+^ T cells were also increased in posterior uveitis patients following treatment with a p55 TNFα receptor fusion protein (37). Earlier studies assessing mouse transgenic T cells (38) or human CD4^+^ T cell clones (39) also found that TNF blockade led to increased IL-10 production in cell culture supernatants. In further support of our data, Boks et al. showed that blockade of TNFα during *in vitro* priming of naïve CD4^+^ T cells with tolerogenic DCs favored the development of IL-10^+^ CD4^+^ T cells (40). Thus, TNFi-mediated regulation of IL-10 in CD4^+^ T cells has been indicated by a growing number of *in vitro* and *ex vivo* studies.

The presence of IL-10-expressing CD8^+^ T cells has been reported in several mouse models of infection, including acute influenza infection (41), chronic *Mycobacterium tuberculosis* infection (42), and coronavirus-induced encephalitis (43). Furthermore, IL-10^+^ CD8^+^ T cells have been found in patients with HIV (44) and chronic hepatitis C infection (45). IL-10 expression in CD8^+^ T cells can be induced by IL-4, either alone (46) or in combination with IL-12 (47), or dexamethasone (48). However, to the best of our knowledge, this is the first report showing that TNF blockade can regulate IL-10 expression in CD8^+^ T cells.

IL-27 can stimulate IL-10 production by various effector CD4^+^ T cell subsets (32, 49, 50). Our data suggest however that the capacity of TNF blockade to affect IL-10 expression in CD4^+^ T cells is not dependent on IL-27. In agreement with other work on human T cells (51, 52), we found that addition of recombinant IL-27 to anti-CD3/CD28-stimulated CD4^+^ T cells resulted in increased IL-10 secretion, although this was not strictly dose-dependent. However, in contrast to these other studies, which used naïve CD4^+^ T cells, our analysis of intracellular cytokine expression did not demonstrate increased IL-10^+^ CD4^+^ T cell frequencies after 3 days in culture with IL-27. This is possibly due to different kinetics of IL-10 expression in a bulk CD4^+^ T cell population.

It is of note that although in CD4^+^ T cell/monocyte cocultures IL-10 blockade led to reduced IL-10^+^ frequencies in both the presence and absence of TNF blockade, IL-10 addition by itself was not sufficient to drive IL-10 expression in CD4^+^ T cells. These data are in line with previous work showing that IL-10 is necessary but not sufficient to enhance development of IL-10-producing Tr1 cells following *in vitro* differentiation in the presence of vitamin D3 plus dexamethasone (53). However, when added to anti-CD3-stimulated whole PBMC cultures, rhIL-10 did slightly increase IL-10^+^ CD4^+^ T cell frequencies after 3 and 5 days in culture, indicating that the regulation of IL-10 expression in CD4^+^ T cells is dependent on *in vitro* culture conditions.

In inflammatory diseases, such as RA, the normal balance of immune regulation is disturbed and T cell-derived pro-inflammatory mediators can contribute to disease pathogenesis in an uncontrolled manner. This study indicates that a potentially immunoregulatory IL-10^+^ phenotype is broadly maintained in effector T cells following exposure to TNF blockade, which may represent an underappreciated mechanism of action for this widely used therapeutic strategy. We previously provided evidence that the IL-10 secreted by IL-17^+^ CD4^+^ T cells following TNF blockade is biologically active (20); however, further work is required to investigate whether the increase in IL-10^+^ frequencies across different T cell subpopulations is functionally relevant in the context of inflammatory disease. Additional insights into the underlying molecular mechanisms *via* which TNF blockade maintains IL-10 expression could identify potential targets for novel therapeutic strategies.

## Author Contributions

CR, LD, and VF designed and performed experiments and analyzed and interpreted data. LT and HE conceived the study, contributed to experimental design, and interpreted data. CR and LT wrote and revised the manuscript. All authors edited the manuscript.

## Conflict of Interest Statement

The authors declare that the research was conducted in the absence of any commercial or financial relationships that could be construed as a potential conflict of interest.

## References

[1] Feldmann M. Development of anti-TNF therapy for rheumatoid arthritis. Nat Rev Immunol (2002) 2:364–71. 10.1038/nri802 12033742

[2] Taylor PC Feldmann M. Anti-TNF biologic agents: still the therapy of choice for rheumatoid arthritis. Nat Rev Rheumatol (2009) 5(10):578–82. 10.1038/nrrheum.2009.181 19798034

[3] Hetland ML Christensen IJ Tarp U Dreyer L Hansen A Hansen IT Direct comparison of treatment responses, remission rates, and drug adherence in patients with rheumatoid arthritis treated with adalimumab, etanercept, or infliximab: results from eight years of surveillance of clinical practice in the nationwide Danish DANBIO registry. Arthritis Rheum (2010) 62(1):22–32. 10.1002/art.27227 20039405

[4] Mariette X Ravaud P Steinfeld S Baron G Goetz J Hachulla E Inefficacy of infliximab in primary Sjögren’s syndrome: results of the randomized, controlled trial of remicade in primary Sjögren’s syndrome (TRIPSS). Arthritis Rheum (2004) 50(4):1270–6. 10.1002/art.20146 15077311

[5] Moutsopoulos NM Katsifis GE Angelov N Leakan RA Sankar V Pillemer S Lack of efficacy of etanercept in Sjögren syndrome correlates with failed suppression of tumour necrosis factor α and systemic immune activation. Ann Rheum Dis (2008) 67(10):1437–43. 10.1136/ard.2007.077891 18198195

[6] The Lenercept Multiple Sclerosis Study Group, The University of British Columbia MS/MRI Analysis Group. TNF neutralization in MS: results of a randomized, placebo-controlled multicenter study. Neurology (1999) 53:457–65. 10.1212/WNL.53.3.457 10449104

[7] Perez-Alvarez R Pérez-de-Lis M Ramos-Casals M BIOGEAS Study Group Biologics-induced autoimmune diseases. Curr Opin Rheumatol (2013) 25:56–64. 10.1097/BOR.0b013e32835b1366 23114587

[8] Arora T Padaki R Liu L Hamburger AE Ellison AR Stevens SR Differences in binding and effector functions between classes of TNF antagonists. Cytokine (2009) 45(2):124–31. 10.1016/j.cyto.2008.11.008 19128982

[9] Kaymakcalan Z Sakorafas P Bose S Scesney S Xiong L Hanzatian DK Comparisons of affinities, avidities, and complement activation of adalimumab, infliximab, and etanercept in binding to soluble and membrane tumor necrosis factor. Clin Immunol (2009) 131(2):308–16. 10.1016/j.clim.2009.01.002 19188093

[10] Mitoma H Horiuchi T Tsukamoto H Tamimoto Y Kimoto Y Uchino A Mechanisms for cytotoxic effects of anti-tumor necrosis factor agents on transmembrane tumor necrosis factor α-expressing cells: comparison among infliximab, etanercept, and adalimumab. Arthritis Rheum (2008) 58(5):1248–57. 10.1002/art.23447 18438840

[11] Nesbitt A Fossati G Bergin M Stephens P Stephens S Foulkes R Mechanism of action of certolizumab pegol (CDP870): in vitro comparison with other anti-tumor necrosis factor alpha agents. Inflamm Bowel Dis (2007) 13(11):1323–32. 10.1002/ibd.20225 17636564

[12] Baldwin HM Ito-Ihara T Isaacs JD Hilkens CM. Tumour necrosis factor alpha blockade impairs dendritic cell survival and function in rheumatoid arthritis. Ann Rheum Dis (2010) 69(6):1200–7. 10.1136/ard.2009.110502 19773288

[13] Nadkarni S Mauri C Ehrenstein MR. Anti-TNF-alpha therapy induces a distinct regulatory T cell population in patients with rheumatoid arthritis via TGF-beta. J Exp Med (2007) 204(1):33–9. 10.1084/jem.20061531 17200409 PMC2118431

[14] Ehrenstein MR Evans JG Singh A Moore S Warnes G Isenberg DA Compromised function of regulatory T cells in rheumatoid arthritis and reversal by anti-TNFα therapy. J Exp Med (2004) 200(3):277–85. 10.1084/jem.20040165 15280421 PMC2211983

[15] Nguyen DX Ehrenstein MR. Anti-TNF drives regulatory T cell expansion by paradoxically promoting membrane TNF-TNF-RII binding in rheumatoid arthritis. J Exp Med (2016) 213(7):1241–53. 10.1084/jem.20151255 27270893 PMC4925013

[16] Blache C Lequerre T Roucheux A Beutheu S Dedreux I Jacquot S Number and phenotype of rheumatoid arthritis patients’ CD4+CD25hi regulatory T cells are not affected by adalimumab or etanercept. Rheumatology (Oxford) (2011) 50(10):1814–22. 10.1093/rheumatology/ker183 21791546

[17] Dombrecht EJ Aerts NE Schuerwegh AJ Hagendorens MM Ebo DG Van Offel JF Influence of anti-tumor necrosis factor therapy (Adalimumab) on regulatory T cells and dendritic cells in rheumatoid arthritis. Clin Exp Rheumatol (2006) 24:31–7. 16539816

[18] Kleijwegt FS Laban S Duinkerken G Joosten AM Zaldumbide A Nikolic T Critical role for TNF in the induction of human antigen-specific regulatory T cells by tolerogenic dendritic cells. J Immunol (2010) 185(3):1412–8. 10.4049/jimmunol.1000560 20574005

[19] Grinberg-Bleyer Y Saadoun D Baeyens A Billiard F Goldstein JD Gregoire S Pathogenic T cells have a paradoxical protective effect in murine autoimmune diabetes by boosting Tregs. J Clin Invest (2011) 120(12):4558–68. 10.1172/JCI42945 PMC2993590 21099113

[20] Evans HG Roostalu U Walter GJ Gullick NJ Frederiksen KS Roberts CA TNF-α blockade induces IL-10 expression in human CD4+ T cells. Nat Commun (2014) 5:3199 10.1038/ncomms4199 24492460 PMC3918582

[21] Roberts CA Dickinson AK Taams LS The interplay between monocytes/macrophages and CD4+ T cell subsets in rheumatoid arthritis. Front Immunol (2015) 6:571 10.3389/fimmu.2015.00571 26635790 PMC4652039

[22] Noack M Miossec P. Th17 and regulatory T cell balance in autoimmune and inflammatory diseases. Autoimmun Rev (2014) 13(6):668–77. 10.1016/j.autrev.2013.12.004 24418308

[23] Strober W Fuss IJ. Proinflammatory cytokines in the pathogenesis of inflammatory bowel diseases. Gastroenterology (2011) 140(6):1756–67. 10.1053/j.gastro.2011.02.016 21530742 PMC3773507

[24] Fletcher JM Lalor SJ Sweeney CM Tubridy N Mills KH T cells in multiple sclerosis and experimental autoimmune encephalomyelitis. Clin Exp Immunol (2010) 162(1):1–11. 10.1111/j.1365-2249.2010.04143.x 20682002 PMC2990924

[25] Srenathan U Steel K Taams LS. IL-17+ CD8+ T cells: differentiation, phenotype and role in inflammatory disease. Immunol Lett (2016) 178:20–6. 10.1016/j.imlet.2016.05.001 27173097 PMC5046976

[26] Petrelli A van Wijk F. CD8(+) T cells in human autoimmune arthritis: the unusual suspects. Nat Rev Rheumatol (2016) 12(7):421–8. 10.1038/nrrheum.2016.74 27256711

[27] Menon B Gullick NJ Walter GJ Rajasekhar M Garrood T Evans HG IL-17+CD8+ T-cells are enriched in the joints of patients with psoriatic arthritis and correlate with disease activity and joint damage progression. Arthritis Rheumatol (2014) 66:1272–81. 10.1002/art.38376 24470327 PMC4158887

[28] Hijnen D Knol EF Gent YY Giovannone B Beijn SJ Kupper TS CD8+ T cells in the lesional skin of atopic dermatitis and psoriasis patients are an important source of IFN-γ, IL-13, IL-17, and IL-22. J Invest Dermatol (2013) 133(4):973–9. 10.1038/jid.2012.456 23223131 PMC3835628

[29] Res PC Piskin G de Boer OJ van der Loos CM Teeling P Bos JD Overrepresentation of IL-17A and IL-22 producing CD8 T cells in lesional skin suggests their involvement in the pathogenesis of psoriasis. PLoS One (2010) 5(11):e14108. 10.1371/journal.pone.0014108 21124836 PMC2991333

[30] Tracey D Klareskog L Sasso EH Salfeld JG Tak PP. Tumor necrosis factor antagonist mechanisms of action: a comprehensive review. Pharmacol Ther (2008) 117(2):244–79. 10.1016/j.pharmthera.2007.10.001 18155297

[31] Chen DY Chen YM Tsai WC Tseng JC Chen YH Hsieh CW Significant associations of antidrug antibody levels with serum drug trough levels and therapeutic response of adalimumab and etanercept treatment in rheumatoid arthritis. Ann Rheum Dis (2015) 74(3):e16. 10.1136/annrheumdis-2013-203893 24442879

[32] Stumhofer JS Silver JS Laurence A Porrett PM Harris TH Turka LA Interleukins 27 and 6 induce STAT3-mediated T cell production of interleukin 10. Nat Immunol (2007) 8(12):1363–71. 10.1038/ni1537 17994025

[33] de Waal Malefyt R Haanen J Spits H Roncarolo MG te Velde A Figdor C Interleukin 10 (IL-10) and viral IL-10 strongly reduce antigen-specific human T cell proliferation by diminishing the antigen-presenting capacity of monocytes via downregulation of class II major histocompatibility complex expression. J Exp Med (1991) 174:915–24. 10.1084/jem.174.4.915 1655948 PMC2118975

[34] Sulahian TH Hogger P Wahner AE Wardwell K Goulding NJ Sorg C Human monocytes express CD163, which is upregulated by IL-10 and identical to p155. Cytokine (2000) 12(9):1312–21. 10.1006/cyto.2000.0720 10975989

[35] Ebert EC Infliximab and the TNF-α system. Am J Physiol Gastrointest Liver Physiol (2009) 296(3):G612–20. 10.1152/ajpgi.90576.2008 19136378

[36] Antiga E Volpi W Cardilicchia E Maggi L Filì L Manuelli C Etanercept downregulates the Th17 pathway and decreases the IL-17+/IL-10+ cell ratio in patients with psoriasis vulgaris. J Clin Immunol (2012) 32:1221–32. 10.1007/s10875-012-9716-x 22699761

[37] Greiner K Murphy CC Willermain F Duncan L Plskova J Hale G Anti-TNFα therapy modulates the phenotype of peripheral blood CD4+ T cells in patients with posterior segment intraocular inflammation. Invest Ophthalmol Vis Sci (2004) 45(1):170–6. 10.1167/iovs.03-0659 14691170

[38] Cope A Ettinger R McDevitt H The role of TNF alpha and related cytokines in the development and function of the autoreactive T-cell repertoire. Res Immunol (1997) 148:307–12. 10.1016/S0923-2494(97)87239-2 9352594

[39] Cope AP Londei M Chu NR Cohen SB Elliott MJ Brennan FM Chronic exposure to tumor necrosis factor (TNF) in vitro impairs the activation of T cells through the T cell receptor/CD3 complex; reversal in vivo by anti-TNF antibodies in patients with rheumatoid arthritis. J Clin Invest (1994) 94:749–60. 10.1172/JCI117394 8040330 PMC296155

[40] Boks MA Kager-Groenland JR Mousset CM van Ham SM ten Brinke A Inhibition of TNF receptor signaling by anti-TNFα biologicals primes naïve CD4+ T cells towards IL-10+ T cells with a regulatory phenotype and function. Clin Immunol (2014) 151(2):136–45. 10.1016/j.clim.2014.02.008 24568737

[41] Sun J Madan R Karp CL Braciale TJ. Effector T cells control lung inflammation during acute influenza virus infection by producing IL-10. Nat Med (2009) 15(3):277–84. 10.1038/nm.1929 19234462 PMC2693210

[42] Cyktor JC Carruthers B Beamer GL Turner J. Clonal expansions of CD8+ T cells with IL-10 secreting capacity occur during chronic *Mycobacterium tuberculosis* infection. PLoS One (2013) 8(3):e58612. 10.1371/journal.pone.0058612 23472214 PMC3589362

[43] Trandem K Zhao J Fleming E Perlman S. Highly activated cytotoxic CD8 T cells express protective IL-10 at the peak of coronavirus-induced encephalitis. J Immunol (2011) 186(6):3642–52. 10.4049/jimmunol.1003292 21317392 PMC3063297

[44] Elrefaei M Barugahare B Ssali F Mugyenyi P Cao H. HIV-specific IL-10-positive CD8+ T cells are increased in advanced disease and are associated with decreased HIV-specific cytolysis. J Immunol (2006) 176(2):1274–80. 10.4049/jimmunol.176.2.1274 16394019

[45] Abel M Sène D Pol S Bourlière M Poynard T Charlotte F Intrahepatic virus-specific IL-10-producing CD8 T cells prevent liver damage during chronic hepatitis C virus infection. Hepatology (2006) 44(6):1607–16. 10.1002/hep.21438 17133491

[46] Zhao Y Zhao H Sun Y Hao J Qi X Zhou X IL-4 induces a suppressive IL-10-producing CD8+ T cell population via a Cdkn2a-dependent mechanism. J Leukoc Biol (2013) 94(6):1103–12. 10.1189/jlb.0213064 23772040 PMC6607996

[47] Noble A Giorgini A Leggat JA Cytokine-induced IL-10-secreting CD8 T cells represent a phenotypically distinct suppressor T-cell lineage. Blood (2006) 107(11):4475–83. 10.1182/blood-2005-10-3994 16467201

[48] Richards DF Fernandez M Caulfield J Hawrylowicz CM. Glucocorticoids drive human CD8(+) T cell differentiation towards a phenotype with high IL-10 and reduced IL-4, IL-5 and IL-13 production. Eur J Immunol (2000) 30:2344–54. 10.1002/1521-4141(2000)30:8<2344::AID-IMMU2344>3.0.CO;2-7 10940925

[49] Fitzgerald DC Zhang GX El-Behi M Fonseca-Kelly Z Li H Yu S Suppression of autoimmune inflammation of the central nervous system by interleukin 10 secreted by interleukin 27-stimulated T cells. Nat Immunol (2007) 8(12):1372–9. 10.1038/ni1540 17994023

[50] Awasthi A Carrier Y Peron JP Bettelli E Kamanaka M Flavell RA A dominant function for interleukin 27 in generating interleukin 10-producing anti-inflammatory T cells. Nat Immunol (2007) 8(12):1380–9. 10.1038/ni1541 17994022

[51] Murugaiyan G Mittal A Lopez-Diego R Maier LM Anderson DE Weiner HL. IL-27 is a key regulator of IL-10 and IL-17 production by human CD4+ T cells. J Immunol (2009) 183(4):2435–43. 10.4049/jimmunol.0900568 19625647 PMC2904948

[52] Wang H Meng R Li Z Yang B Liu Y Huang F IL-27 induces the differentiation of Tr1-like cells from human naive CD4+ T cells via the phosphorylation of STAT1 and STAT3. Immunol Lett (2011) 136(1):21–8. 10.1016/j.imlet.2010.11.007 21115047

[53] Barrat FJ Cua DJ Boonstra A Richards DF Crain C Savelkoul HF In vitro generation of interleukin 10-producing regulatory CD4+ T cells is induced by immunosuppressive drugs and inhibited by T helper type 1 (Th1)- and Th2-inducing cytokines. J Exp Med (2002) 195(5):603–16. 10.1084/jem.20011629 11877483 PMC2193760

