# Supplementary material for: TNF Blockade Maintains an IL-10+ Phenotype in Human Effector CD4+ and CD8+ T Cells
Source: Front Immunol. 2017 Feb 15;8:157. doi: 10.3389/fimmu.2017.00157 (PMC5309392; doi:10.3389/fimmu.2017.00157)
Supplement: Supplementary file 1 [file presentation_1.pdf]

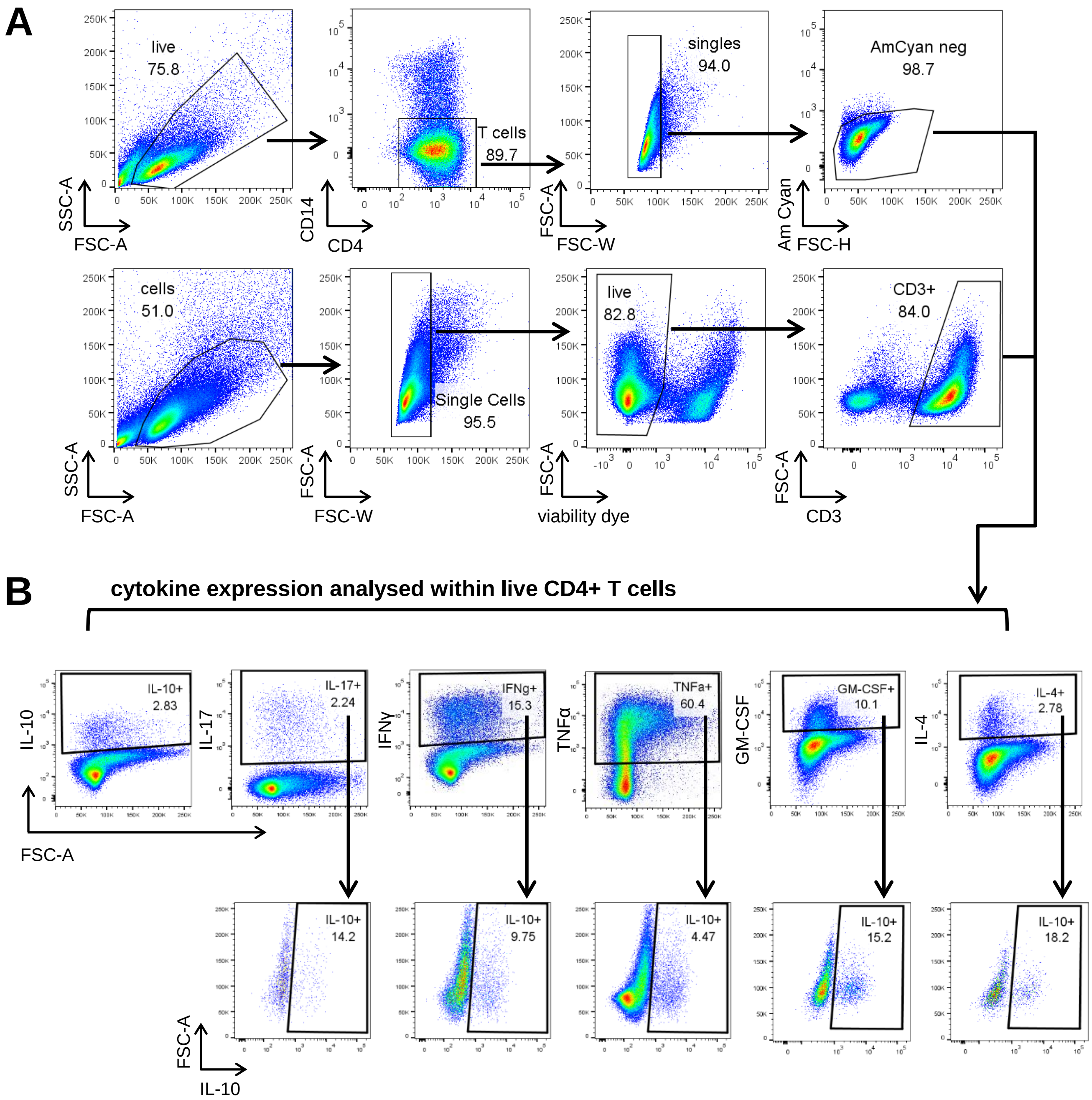

Supplementary Figure 1. Gating strategy for analysis of cytokine expressing cells following CD4+ T cell/CD14+ monocyte co-cultures. (A) A live gate was set around viable lymphocytes based on forward scatter/side scatter (FSC/SSC). Within CD14- CD4+ cells, doublets were excluded. In some experiments AmCyan negative events were gated to reduce interference from cellular autofluorescence (upper row), or where a fixable viability dye was included, this was plotted against FSC and used to exclude any remaining dead cells, and CD3 was stained intracellularly to identify T cells (lower row). **(B)** Within live CD4+ T cells each cytokine was plotted individually against FSC and gates were placed using a sample stained without anti-cytokine antibody. IL-10+ cells were then analyzed as a proportion of total CD4+ T cells or within the IL-17+, IFNγ+, TNFα+, GM-CSF+ or IL-4+ CD4+ T cell subsets, as indicated.

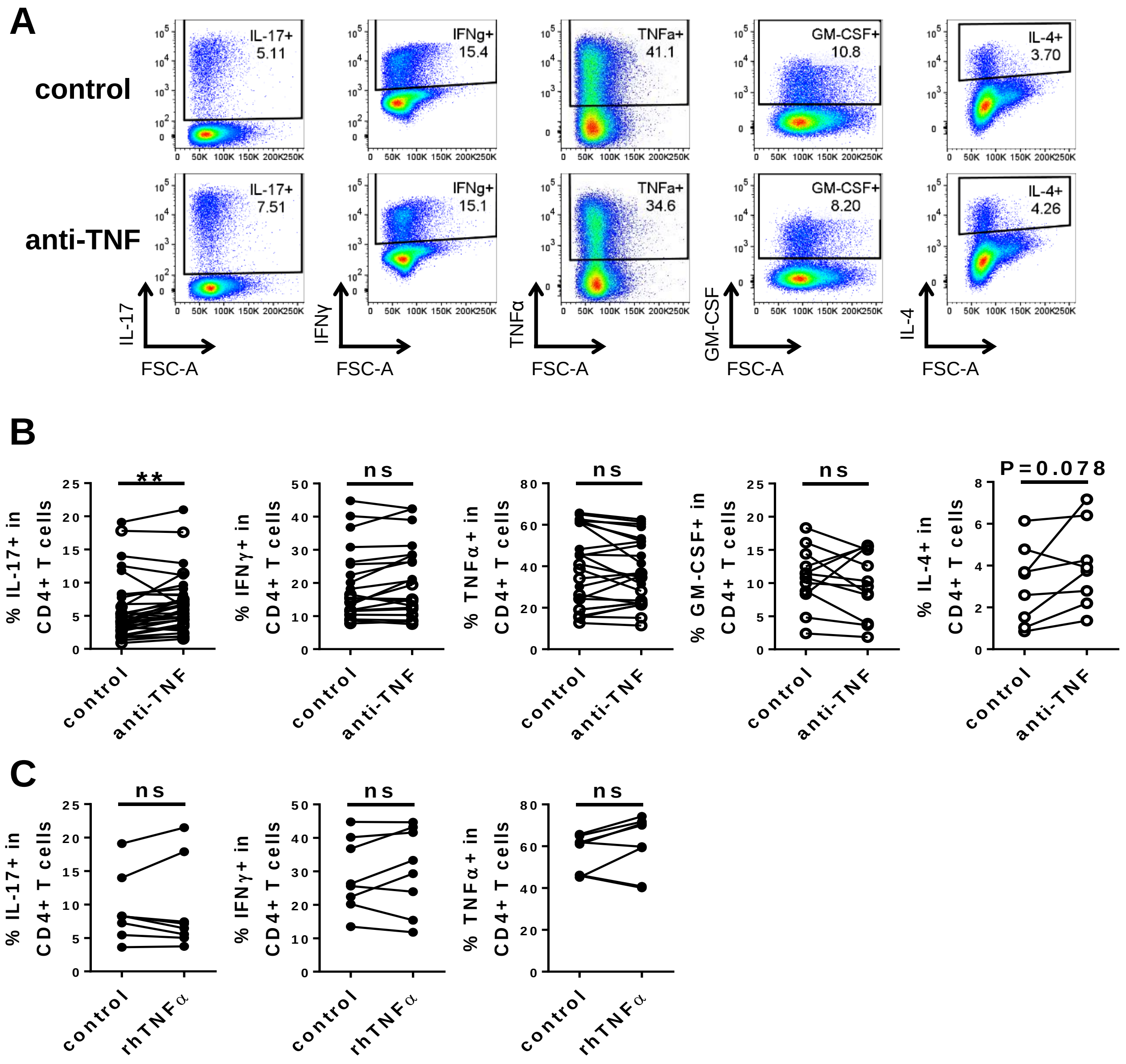

Supplementary Figure 2. Effects of TNF blockade and hrTNFα addition on total IL-17+, IFNγ+, TNFα+, GM-CSF+ or IL-4+ CD4+ T cell populations. CD4+ T cells (open symbols) or CD4+CD45RO+ T cells (black symbols) were co-cultured with CD14+ monocytes and anti-CD3 mAb in the absence or presence of adalimumab (1 µg/ml) (**A, B**) or in the presence of hrTNFα (10 ng/ml) (**C**) for three days as described in the methods. CD4+ T cells were gated as described in Supplementary Figure 1. **(A, B)** Representative dot plots (**A**) and cumulative data (**B**) showing the percentages of IL-17+ (*n* = 35), IFNγ+ (*n* = 21), TNFα+ (*n* = 24), GM-CSF+ (*n* = 12) or IL-4+ (*n* = 8) cells within CD4+ T cells in the absence or presence of anti-TNF. **(C)** Cumulative data showing the percentages of IL-17+ (*n* = 8), IFNγ+ (*n* = 8) or TNFα+ (*n* = 8) cells within CD4+CD45RO+ T cells in the absence or presence of hrTNFα. Data were analyzed using Wilcoxon matched-pairs signed rank test. Each connecting line represents an individual donor. ns p>0.05, ** p<0.01.

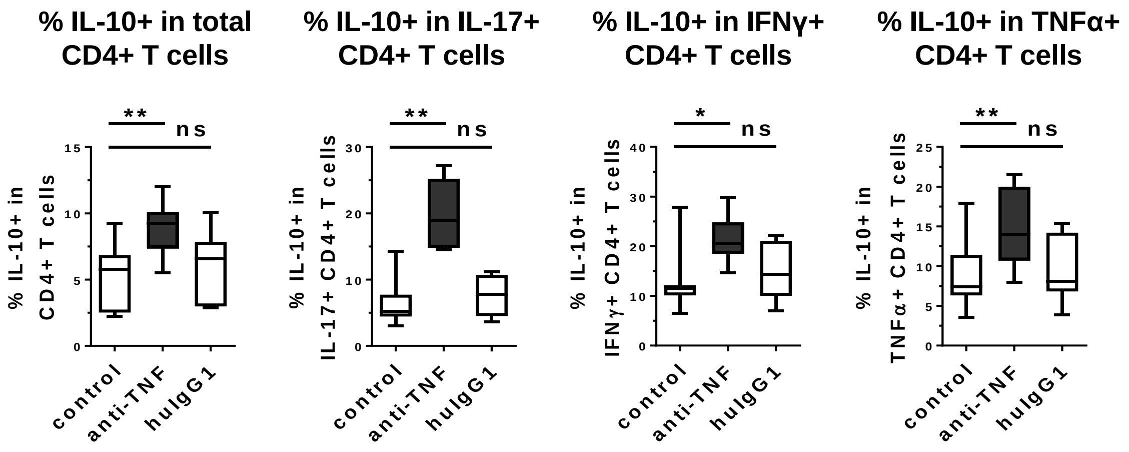

Supplementary Figure 3. Isotype control human IgG mAb does not promote IL-10 expression in CD4+ T cells. CD4+ T cells (n = 3) or CD4+CD45RO+ T cells (n = 4) were co-cultured with CD14+ monocytes and anti-CD3 mAb in the absence or presence of adalimumab (1 µg/ml) or human IgG mAb (1 µg/ml) for three days as described in the methods. CD4+ T cells were gated as described in Supplementary Figure 1 and the percentage of IL-10+ cells within total CD4+, or within IL-17+, IFNγ+ or TNFα+ CD4+ T cells was assessed by flow cytometry. Box-whisker plots show data from n = 7 individual donors; whiskers show minimum and maximum values. Data were analyzed using Friedman test with Dunn's Multiple Comparisons test. ns $p>0.05$, * $p<0.05$, ** $p<0.01$.

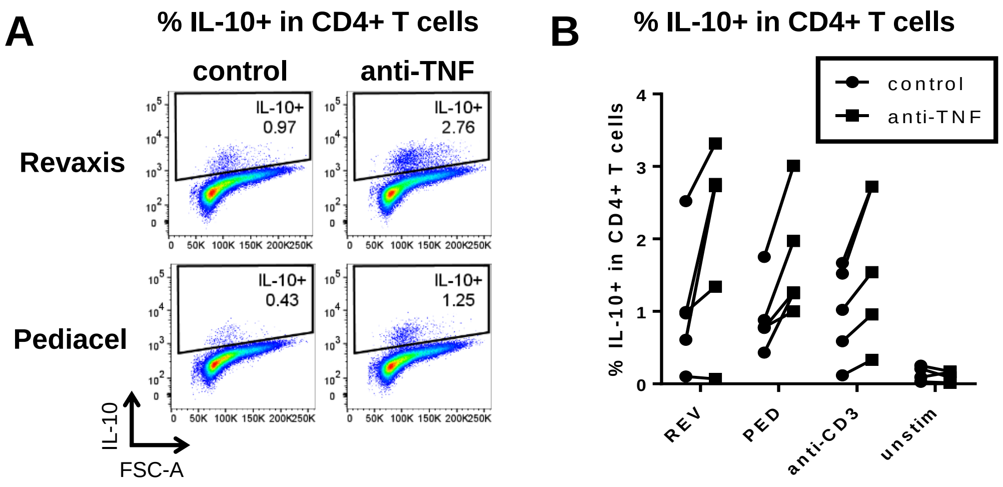

Supplementary Figure 4. TNF blockade promotes IL-10 in CD4+ T cells stimulated in an antigen-specific manner. CD4+ T cells (5 x 10^5) were co-cultured with autologous CD14+ monocytes (5 x 10^4) and stimulated with anti-CD3 mAb (100 ng/ml), Revaxis (REV) or Pediacel (PED) vaccine (both used at 1:1000 dilution) or left unstimulated (unstim), in the absence (control) or presence of anti-TNF mAb (adalimumab, 1 μg/ml). After 6 days cells were re-stimulated with PMA/ionomycin and assessed for intracellular cytokine expression as described in the methods. **(A)** Representative dot plots and **(B)** cumulative data (*n* = 5 except unstim, *n* = 4) showing the frequencies of IL-10+ cells within total CD4+ T cells in the absence or presence of anti-TNF. Each connecting line represents an individual donor.

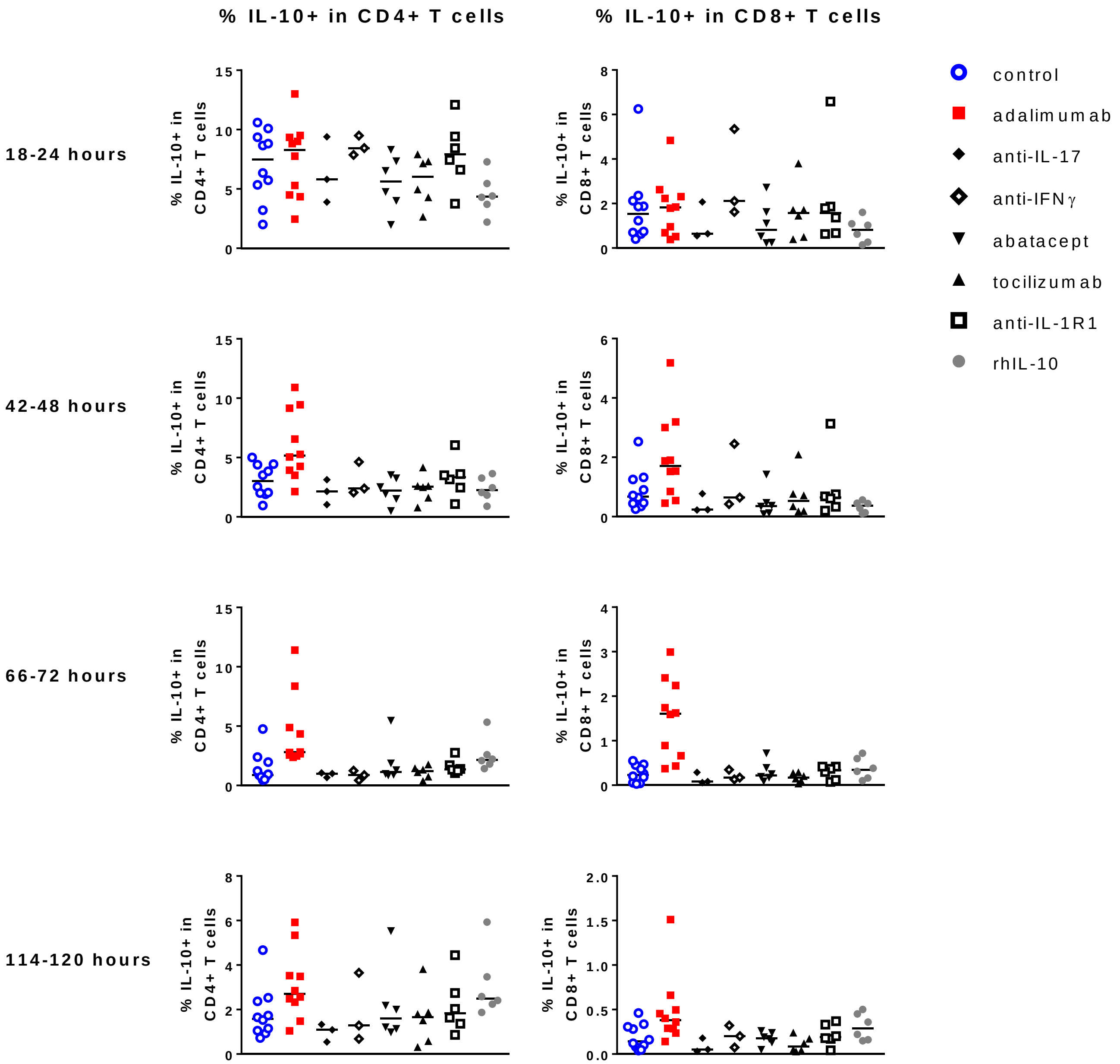

Supplementary Figure 5. Kinetics of IL-10 regulation in CD4+ and CD8+ T cells in response to blockade of various pathways or exogenous recombinant human IL-10. PBMC were cultured in the presence of anti-CD3 mAb alone (control, 100 ng/ml, *n* = 10), or with anti-TNF (adalimumab, 1 μg/ml, *n* = 10), neutralizing antibodies to IFNγ or IL-17 (10 μg/ml, both *n* = 3), tocilizumab (50 μg/ml, n=6), abatacept (5 μg/ml, *n* = 6), anti-IL-1R1 blocking Ab (2 μg/ml, *n* = 6) or recombinant human IL-10 (10 ng/ml, *n* = 6). At various time points (18-120 hours) cells were re-stimulated and assessed for intracellular cytokine expression as described in the methods. Scatter plots represent data showing the effect of TNF blockade on IL-10+ frequencies in CD4+ and CD8+ T cells over time; bars indicate median values.

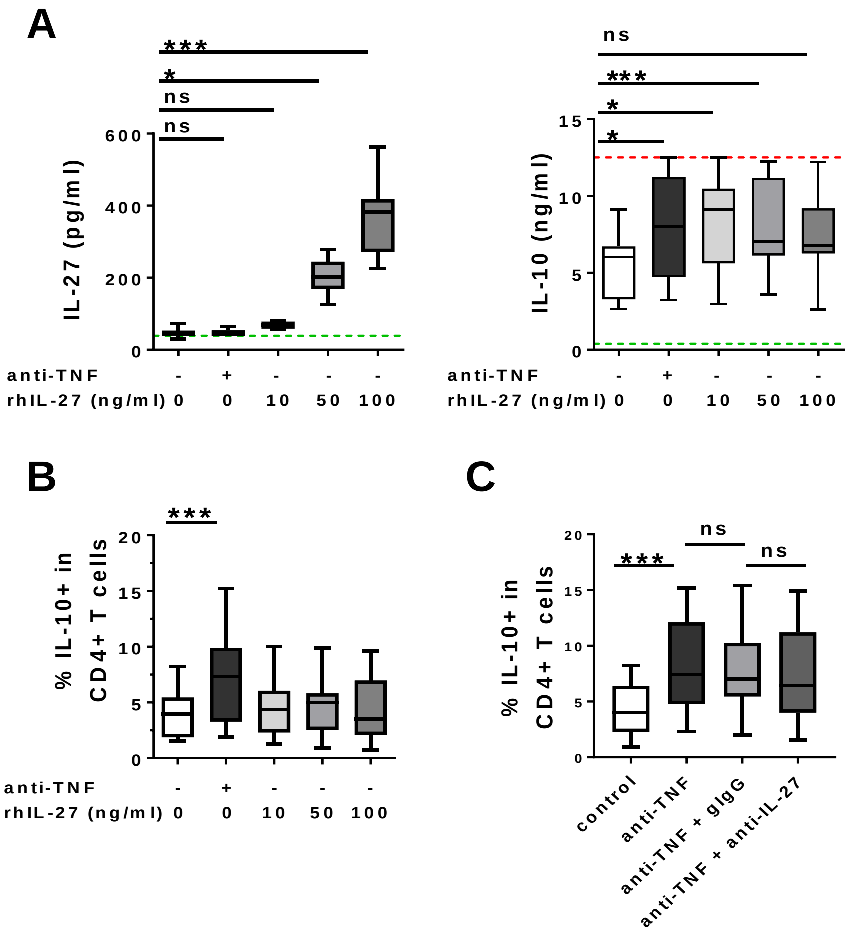

Supplementary Figure 6. The monocyte-derived anti-inflammatory mediator IL-27 does not contribute to IL-10 regulation by TNF blockade. (A, B) CD4+ T cells were stimulated with immobilised anti-CD3 (1.25 μg/ml) and soluble anti-CD28 (1 μg/ml), with or without anti-TNF mAb (adalimumab, 1 μg/ml) or IL-27 (10-100 ng/ml). **(A)** After three days IL-27 and IL-10 levels in culture supernatants were measured by ELISA (*n* = 7), before cells were re-stimulated. **(B)** After three days, cells were assessed for intracellular cytokine expression as described in the methods. Box-whisker plots represent data from *n* = 8 individual donors and show frequencies of IL-10+ cells within the total CD4+ T cell population; whiskers show minimum to maximum values. **(A, B)** Data were assessed by Friedman test with comparison to control by Dunn's multiple comparison test. **(C)** Bulk CD4+ T cells (*n* = 7) or CD45RO+ enriched CD4+ T cells (*n* = 3) were stimulated with immobilised anti-CD3 (1.25 μg/ml) and soluble CD28 (1 μg/ml), or were co-cultured with monocytes and soluble anti-CD3 (100 ng/ml), in the absence (control) or presence of anti-TNF (adalimumab, 1 μg/ml), with or without neutralising anti-IL-27 or goat IgG isotype control (gIgG; both 5 μg/ml). After 3 days cells were assessed for intracellular cytokine expression as described in the methods. Data were analyzed by repeated measures ANOVA followed by Sidak's multiple comparison test. ns $p>0.05$, * $p<0.05$, ** $p<0.01$, *** $p<0.001$.
